# Carbon stocks in Norwegian eelgrass meadows across environmental gradients

**DOI:** 10.1038/s41598-024-74760-3

**Published:** 2024-10-24

**Authors:** Karine Gagnon, Jonas Thormar, Stein Fredriksen, Maria Potouroglou, Jon Albretsen, Hege Gundersen, Kasper Hancke, Eli Rinde, Cecilie Wathne, Kjell Magnus Norderhaug

**Affiliations:** 1 Institute of Marine Research (IMR), His, Norway; 2 Department of Biosciences, University of Oslo, Oslo, Norway; 3 GRID-Arendal, Arendal, Norway; 4 Norwegian Institute for Water Research (NIVA), Oslo, Norway; 5 Norwegian Blue Forests Network (NBFN), Arendal, Norway

**Keywords:** *Zostera marina*, Seagrass, Blue carbon, Carbon sequestration, Ecosystem services, Biogeochemistry, Ecosystem services, Carbon cycle

## Abstract

Seagrass meadows are well-known for their capacity to capture and store blue carbon in sediments. However carbon stocks vary significantly between meadows, spanning more than three orders of magnitude on both local and global scales. Understanding the drivers of seagrass carbon stocks could help improve strategies for incorporating blue carbon into management plans. Here, we measured sediment carbon stocks in eelgrass (*Zostera marina*) meadows and unvegetated areas along the Norwegian coast, spanning wide gradients in temperature, wave exposure, water depth, salinity, and eelgrass biomass. Carbon stocks were generally higher in eelgrass meadows than in adjacent unvegetated areas, yet they displayed considerable variation (400 − 30 000 g C m^−2^ at 50 cm sediment depth) even among nearby sites. Overall, the highest carbon stocks were found in deeper, muddier, sheltered meadows near river mouths. These sites likely have the highest input and retention of carbon from different sources. Consequently, they should be prioritized as conservation targets for preserving coastal blue carbon stocks. Despite ever-increasing efforts to quantify seagrass blue carbon globally, high uncertainties still persist, partly due to differing methodologies, processes, and environmental context. Blue carbon stock estimates could be improved through the coordination of standardised mapping and sampling methods.

**Supplementary Information:**

The online version contains supplementary material available at 10.1038/s41598-024-74760-3.

## Introduction

Coastal vegetated ecosystems such as seagrass meadows, macroalgal forests, salt marshes, and mangrove forests play a fundamental role in carbon sequestration and storage, and the term ‘blue carbon’ has been coined to describe the carbon stored and sequestered by these ecosystems^1,2^.Though concentrated in shallow coastal waters and only covering a small fraction of the ocean floor, these ecosystems often hold very high sediment carbon stocks and could play a potentially significant role in mitigating climate change^2–4^. Seagrass meadows form important carbon sinks with a spatial distribution covering all continents from tropical to subpolar climates, extending up to Arctic latitudes. Compared to the amount sequestered in sediments, the carbon stored in the living biomass of a seagrass meadow is generally relatively low^5^. However, both the above- and belowground living components of seagrasses contribute to their ability to sequester and store large amounts of carbon. The aboveground canopy reduces water flow and wave energy, facilitating the capture and retention of organic particles and detritus, which originates not only from within the seagrass meadow, but also from external sources such as phytoplankton, seston, macroalgae, mangroves, and terrestrial and riverine sources^6–10^. At the same time, the dense belowground structure of roots and rhizomes enables higher retention of particles by reducing sediment resuspension^11–14^.

Global total seagrass carbon stocks are estimated to range between 4 and 9 × 10^3^ Mt (megatonnes = 10^12^ g)^2,5^. However, the carbon stocks found in individual seagrass meadows are highly variable, spanning more than three orders of magnitude, on both local and global scales^15,16^. This variability is dependent on various factors, such as the seagrass species composition in the meadow and the prevailing environmental conditions. Key environmental factors include sediment type, temperature, water depth, current dynamics, wave exposure, turbidity, and salinity^15–20^. Despite an ever-increasing number of studies focused on quantifying the blue carbon stocks in seagrass ecosystems and other similar systems, important knowledge gaps still remain. These include challenges in quantifying the global extent and coverage of seagrass meadows (with estimates ranging from 177 000 to 600 000 km^2^)^21^, the lack of baseline blue carbon stock values in many areas, and high uncertainties in the global rate of seagrass losses and the proportion of their stored carbon released back into the atmosphere^2^. Consequently, estimating the current and future carbon stocks of seagrass meadows, as well as the climate mitigation potential of seagrass protection and restoration, is inherently difficult. These uncertainties pose significant challenges in incorporating blue carbon into management plans and assessing the effectiveness of management and restoration initiatives^2,22^. Given the substantial variability in carbon stock values, it is essential to understand the factors influencing carbon stocks in seagrass meadows under different environmental condition in order to predict their carbon stocks at different spatial scales, and to identify management actions needed to enhance their sequestration rates and preserve carbon stocks. Such interventions may include reducing nutrient inputs^23^ or reintroducing top predators^24^, as well as increasing the spatial coverage of seagrass meadows through targeted conservation and restoration efforts^25^.

Although there have been improvements in the ecological status and distribution of seagrass meadows, mainly due to effective management actions that have reduced nutrient inputs as well as successful restoration efforts^26^, the overall global trend for seagrass meadows remains one of continuous decline^27^. While the ability of marine ecosystems to store and sequester carbon has been known for decades, the interest in protecting and restoring these habitats has rapidly increased in recent years, with growing awareness of their potential importance in mitigating global climate change and maintaining biodiversity^28,29^. The 2022 UN Climate Change Conference (COP 27) final declaration included, for the first time, a dedicated sub-section specifically addressing the ocean. Similarly, the Kunming-Montreal Global Biodiversity Framework commits signatories to effectively conserve and manage at least 30% of marine and coastal areas by 2030, prioritizing areas of importance for biodiversity and ecosystem functioning and services, as well as to effectively restore at least 30% of degraded marine and coastal ecosystems. The framework also highlights the importance of nature-based solutions in minimising the impact of climate change and ocean acidification. Furthermore, the *2013 Supplement to the 2006 IPCC Guidelines for National Greenhouse Gas Inventories: Wetlands* enables countries to include mangrove forests, tidal marshes, and seagrass meadows into their plans to fulfil the objectives of the Paris Agreement under the United Nations Framework Convention on Climate Change. As a result, many new and updated Nationally Determined Contributions (NDCs) now include ocean-based actions, with several explicitly incorporating seagrass in their mitigation actions^30,31^. While several countries are in the process of updating their national inventories to include blue carbon^32^, seagrass has not yet been included in the NDCs of Norway or other Nordic countries^33^.

In this study, we assess sediment carbon stocks and their variability in eelgrass (*Zostera marina*) meadows along the Norwegian coast, using sediment cores collected over several different sampling efforts. Eelgrass is among the most widespread seagrass species, found across the northern hemisphere, spanning from subtropical (24°N) to polar (71°N) latitudes^34,35^. As a fast-growing foundation species, eelgrass forms dense meadows in both intertidal and subtidal soft-sediment areas^34^. In a previous global assessment of 54 meadows, carbon stocks in the top 25 cm of eelgrass bed sediments showed considerable variation, ranging from 300 to 26 500 g C m^−2^ across the northern hemisphere^16^. Several studies have identified sediment characteristics, such as grain size and mud content, as key drivers of the carbon stock capacity in eelgrass meadows^15,17,18^, as well as hydrodynamic variables^16,36^. However, characteristics of the eelgrass itself, such as biomass and shoot density, also play a role^16,37–39^. Despite the wide latitudinal range of eelgrass, the majority of studies on carbon stocks in this species have focused on meadows in temperate latitudes (35–60°N).

Norwegian eelgrass meadows are of particular interest for studying the carbon stocks of this species, because they occur along a wide latitudinal gradient spanning cold temperate to polar latitudes (58°–71°N), i.e. a relatively understudied area of eelgrass distribution, which also span wide gradients of wave exposure, salinity, and depth^40^. The total Norwegian eelgrass sediment carbon stock has been coarsely estimated at 0.25 Mt C, an estimate derived from multiplying the average sediment carbon stock values for the Nordic region by the estimated areal coverage of Norwegian eelgrass meadows (90 km^2^)^40^. However, prior to this study, carbon stocks have only been measured within a single eelgrass meadow in Norway^16^, and thus far the Norwegian carbon accounting system does not include any marine ecosystems^41^. The main objectives of this study were to: (1) provide in situ estimates of carbon stocks in eelgrass meadows along the Norwegian coast, and compare them with adjacent unvegetated sediments, (2) explore the environmental variables driving variability in these carbon stocks, and (3) discuss these results within both the regional and global blue carbon context.

## Methods

### Study area

**Fig. 1:**
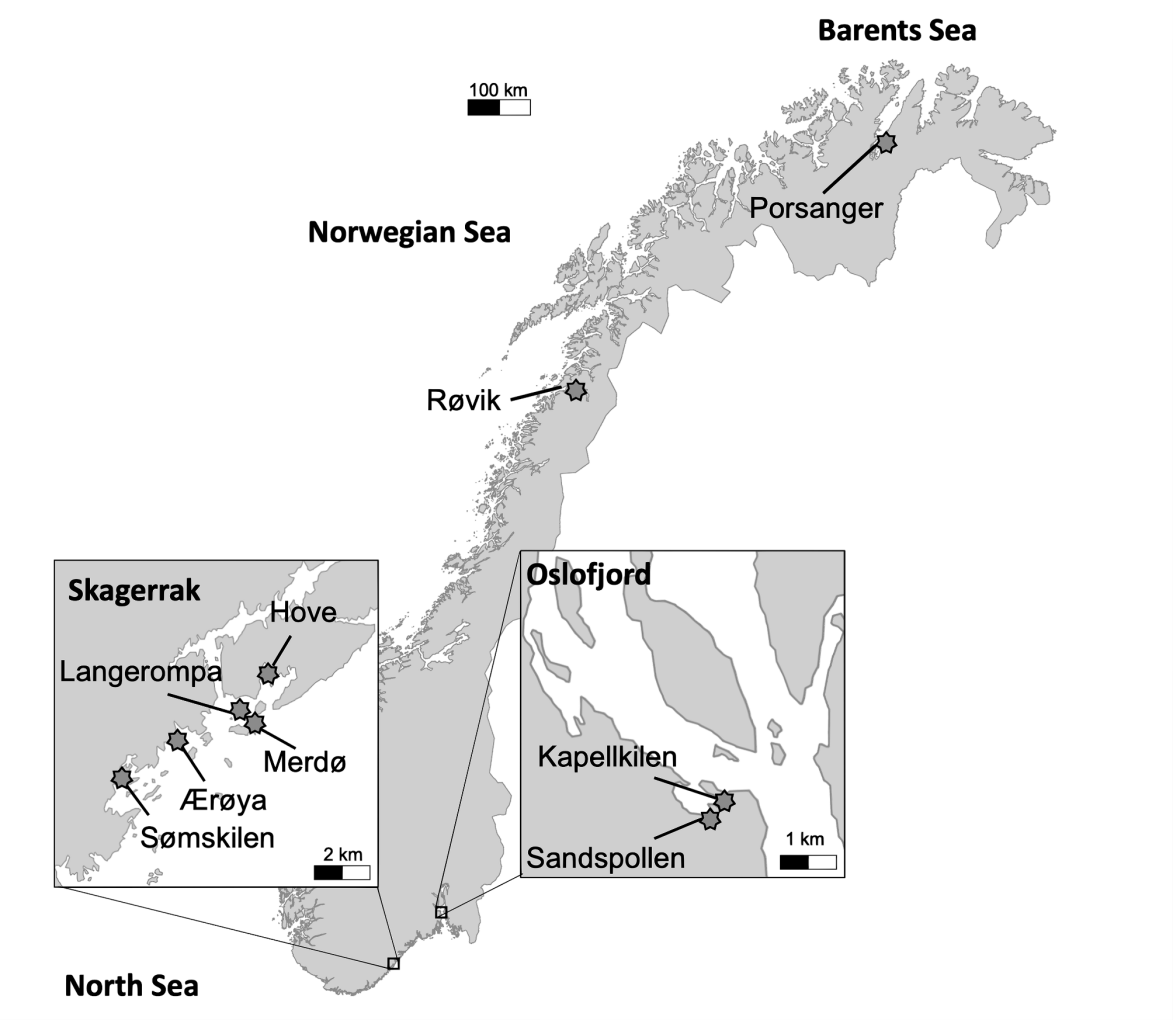
Map of Norway with the nine sampling sites (blue stars) included in the study, distributed within the Barents Sea, Norwegian Sea, and two regions of the North Sea: Skagerrak and Oslofjord. Figure created in QGIS (v3.34.10 ‘Prizren’; www.qgis.org).

This study includes data compiled from several sampling events and projects, leading to minor differences in sampling approaches between sites and years (Table 1). Our study includes data from nine distinct sites across four different regions (Fig. 1). Most of the data originates from five sites in the Skagerrak, southern Norway (Sømskilen, Ærøya, Merdø, Langerompa, and Hove), where samples were taken from both eelgrass meadows and unvegetated sediment in 2017. Additional samples were collected at two sites in the Oslofjord during 2017–2018. These were Sandspollen (unvegetated sediment only) and Kapellkilen (eelgrass and unvegetated sediment). Finally, we incorporated data from two other sampling efforts: one site in the Norwegian Sea in 2015 (Røvik, eelgrass only, previously published^16^), and one site in the Barents Sea in 2022 (Porsanger, eelgrass only). This assemblage of samples allowed us to analyse variation in carbon stocks on both small (within a specific region of the Skagerrak) and large geographic scales (covering four sea areas over the entire Norwegian eelgrass distribution).

**Table 1:** Overview of sampling details and efforts at the nine sites included in the study, in geographic order from south to north. Note that the water depth in Porsanger is given as 0.2 m, but this site is intertidal (all others are subtidal). See table S1 for detailed information on corers, coring depths, and core compression.

Site name	Area	Year	Water depth (m)	Habitat	Sediment type	Replicates
Sømskilen	Skagerrak	2017	3	Eelgrass	Muddy sand	3 + 1 deep core
Sømskilen	Skagerrak	2017	3	Unvegetated	Muddy sand	3
Ærøya	Skagerrak	2017	3	Eelgrass	Coarse sand/gravel	2
Ærøya	Skagerrak	2017	3	Unvegetated	Coarse sand/gravel	3
Merdø	Skagerrak	2017	3	Eelgrass	Sand	3
Merdø	Skagerrak	2017	3	Unvegetated	Sand	3
Langerompa	Skagerrak	2017	3	Eelgrass	Mud	2
Langerompa	Skagerrak	2017	3	Unvegetated	Coarse sand/gravel	3
Langerompa	Skagerrak	2017	7.5	Eelgrass	Mud	3
Langerompa	Skagerrak	2017	7.5	Unvegetated	Coarse sand/gravel	2
Hove	Skagerrak	2017	3	Eelgrass	Fine mud	4 + 1 deep core
Hove	Skagerrak	2017	3	Unvegetated	Mud	2
Sandspollen	Oslofjord	2018	3.7	Unvegetated	Muddy sand	1
Sandspollen	Oslofjord	2018	6	Unvegetated	Muddy sand	1
Kapellkilen	Oslofjord	2017	1.5	Eelgrass	Muddy sand	1
Kapellkilen	Oslofjord	2017	2.5	Eelgrass	Muddy sand	2
Kapellkilen	Oslofjord	2018	3	Eelgrass	Muddy sand	1
Kapellkilen	Oslofjord	2018	6	Unvegetated	Muddy sand	1
Røvik	Norwegian Sea	2015	1	Eelgrass	Sand	3
Porsanger	Barents Sea	2022	0.2	Eelgrass	Coarse sand/gravel	3

### Sediment core sampling

The sampling procedures followed the general methods described in previous global eelgrass carbon sampling efforts. However, as we used cores originating from several different sampling efforts and projects, there were differences between years and sites in terms of replicates, sampling depth, core length and diameter, etc., depending on the environmental characteristics of each site (e.g. sediment type, water depth) and the aims/objectives of the respective projects^16^. At each site, 1–4 sediment cores were collected from either eelgrass meadows, unvegetated areas, or both (see Table 1 for sampling details in the different sites) by SCUBA diving, snorkelling, or wading, depending on the water depth. Sediment cores collected from unvegetated areas were taken at a distance of 10–20 m from an adjacent eelgrass meadow. The site in Porsanger was intertidal, while the others were subtidal, ranging from 1 to 7.5 m depth. Three sites (Langerompa, Sandspollen, and Kapellkilen) were sampled at several different depths (see Table 1 for details). Acrylic corers, with varying lengths (25–100 cm) and diameters (52–56 mm), were pushed into the sediment down to a maximum penetration depth of 6 to 100 cm, depending on the site (see Table S1 for details). Our general goal was to collect sediment to a depth of at least 30–50 cm, however, at some sites with extremely coarse sediment this was not feasible, and we collected sediment to the maximum achievable depth. The cores were securely sealed at both ends while still submerged under water, and then carefully transported upright to the laboratory. Upon arrival, most of the cores were immediately processed in the laboratory, except for those from the Oslofjord sites, which were first sliced and then frozen, and those from Porsanger, which were frozen and subsequently thawed prior to slicing and further processing.

### Deep core sampling

At two sites (Sømskilen and Hove), we collected additional deep cores to explore carbon content beyond the top 50 cm. At Sømskilen, a 200-cm long corer was pushed down to 200 cm depth, (resulting in a 110-cm long sediment core, see Table S1), which was subsequently processed and analysed (see below). At Hove, we used a two-step coring process, where first a 100-cm long corer was pushed to 100 cm depth, and the resulting sediment core discarded. This was done to reduce friction within the subsequent corer. Then, a 200-cm long corer was used in the same hole, reaching an approximate sampling depth of 200 cm. The sediment core obtained from the second extraction (110-cm long) was then processed and analysed (see below), and we assumed that the bottom portion of this core contains sediment from 200 cm depth and upwards.

### Laboratory analyses

In the laboratory, the cores were sectioned for analysis. For most of the cores, the top 20 cm were sliced into 2-cm thick slices, and the remaining part of the core into 5-cm thick slices. This was done because several previous studies have shown that carbon content can vary significantly in the top sections of the sediment, while the deeper sections are usually more stable^18,42^. The deep cores were sliced into 10-cm thick slices from 20 to 110 cm (Sømskilen) and from 60 to 110 cm (Hove), as we were especially interested in the carbon contents of the deeper layers of these cores. Thus the total number of sediment slices analysed amounted to 373, with an additional 18 slices coming from the deep cores). Eelgrass rhizomes and roots, as well as any visible large animals (e.g. bivalves) were removed from the sediment after slicing. From each sediment slice, a subsample comprising a known volume (20–50 ml) was dried at 60 °C for 48–72 h to calculate dry bulk density (DBD, g cm^−3^). This subsample was then combusted at 450 °C for 4 h to calculate the sediment organic content through loss on ignition (LOI). A second subsample of approximately 1 g was taken from a random selection of 96 slices from the 2017 Skagerrak sites, which were finely ground with a mortar and pestle to remove aggregates, and sent to SUERC (www.gla.ac.uk/research/az/suerc/*)* for analysis of total organic carbon (TOC) content and stable isotopes (δ^13^C) in each sample. ^13^C analyses of these samples were undertaken using a VG SIRA 11 IRMS, comparing sample values with those of a working standard reference gas of known isotopic composition produced from international reference materials NBS19 and IAEA-CO-1. The measurement results are expressed using the δ notation^43^ as per mil deviations from the VPDB standard, with 1σ precision of ± 0.1‰:

δ^13^C = (R_sample_/R_standard_ -1) x 1000, where R is the isotope ratio of 13 C:12 C.

We analysed δ^13^C, as it is a useful indicator of the relative contribution of eelgrass-derived carbon versus carbon from other sources in the sediment, such as macroalgae, phytoplankton, and particulate organic matter. In general, eelgrass has higher (-6 to -11) δ^13^C values than macroalgae (-14 to -24) and particulate organic carbon in the water column (-20 to -25)^44–48^. Therefore, higher δ^13^C values in the sediment could potentially indicate a greater proportion of carbon derived from eelgrass.

### Organic carbon stock calculations

Loss on ignition (LOI) and total organic carbon (TOC) are closely linked, and TOC can be approximated from LOI values based on regression analysis, thus reducing analysis efforts and costs^5^. Based on an analysis of the 95 subsamples, we derived the following regression equation, which shows a strong correlation (R^2^ = 0.95):

$$\% {\text{TOC}} = \left( 0.4903 \times \% {\text{LOI}} \right) - 0.394$$

We then used this equation to predict TOC for the remaining samples with LOI > 1%. However, the relationship between LOI and TOC becomes less predictable at very low carbon levels, and using the general regression equation above would have resulted in negative carbon values. We therefore calculated a separate regression equation based on 16 subsamples with LOI < 1%, and used this to predict organic carbon content for the remaining low LOI-samples (which included most samples from the Ærøya, Merdø, and Porsanger sites):

$$\% {\text{TOC}} = \left( 0.2283 \times \% {\text{LOI}} \right) - 0.026$$

Despite the higher uncertainty at low carbon levels, its impact on the overall carbon stock calculations is negligible due to the minimal contribution from these sites. Overall, given the high correlation between LOI and organic carbon, along with the fact that organic carbon makes up more than 95% of the carbon content in Nordic eelgrass sediments^18^, we consider this approach to provide a reliable estimate of the total carbon content.

To determine carbon density (g C m^−3^), we multiplied the organic carbon content by the dry bulk density (DBD) of each slice. From these values, we constructed vertical sediment profiles of each core to visualize the carbon density throughout the whole length of the core. We then calculated the carbon content (g C m^−2^) of each slice by multiplying the carbon density by the thickness of the slice, and summed the carbon content of all slices to obtain the carbon stock per square meter of each core. To enable comparisons across cores and sites, we inter/extrapolated carbon stocks for the top 50 cm of sediment, adjusting for the total length of each core. For the previously published data from Røvik, where carbon stocks were reported from the top 25 cm of the compressed core length, we doubled these values to standardize the measurements across all cores. We used these estimates of carbon stock from the top 50 cm for all further analyses and figures, with the exception of the sediment depth profiles, where the original, unadjusted data are presented.

When using open-barrel corers (as we did) in soft sediments, the resulting sediment core will often be shorter in length than the penetration depth - a phenomenon known as core shortening. This discrepancy is due to a variety of opposing forces, including the friction between the sediment and the internal surface of the corer^49^. As resistance increases when the corer is pushed deeper, some of the sediment is pushed aside (rather than being compacted) leading to smaller increases in the sample’s length with each additional centimeter of depth, until a point when it is effectively blocked^49^. The coring itself is not expected to alter sediment density, although transportation and slicing may cause small discrepancies. Core shortening can affect the vertical profiles of all sediment parameters so that e.g. a 5 cm slice from the 30–35 cm section of the core may actually consist of sediment from 30 to 50 cm depth. If not corrected for, comparison of different cores can be biased, as can carbon stock estimates and extrapolations. However, proper correction is complicated as the functional relationship between penetration depth and length of the sediment core is unique and non-linear for each combination of sediment composition and corer parameters (e.g. diameter). Some studies apply a linear correction to sediment depth and dry bulk density to limit biases^50–52^. Such correction may provide a better representation of the vertical profile than uncorrected cores, but may equally introduce errors in both profiles and stocks when core shortening is high and vertical profile parameters change with depth. In this study we did not correct for shortening. This will result in vertical profiles that in the upper half of the penetration depth will better represent the true sediment conditions than linear correction but with higher deviance in the lower and increasingly shortened part of the core. However, for reference, we also document the penetration depths and the sediment core lengths prior to slicing (Table S1). We further address the limitations and uncertainties associated with core sampling depth in the Discussion section.

### Eelgrass biomass and environmental data

We collected environmental data relating to the eelgrass characteristics, sediment characteristics, water properties, and temperature (see Table 2 for details and sources and Table S2 for an overview of the environmental variables in all sites). From each eelgrass meadow, except for Kapellkilen and Porsanger, we collected 2–3 additional cores (diameter: 10–15 cm) from which we sorted aboveground and belowground eelgrass material and calculated the dry weight biomass from each, after drying at 60 °C for 48 h. For Porsanger, we estimated the eelgrass biomass from the sediment cores themselves after thawing.

For all sites, we calculated the wave exposure index using a model based on fetch and historical wind strength in 16 directions, with a spatial resolution of 25 m. This model, which has been developed for the entire Norwegian coast, has been widely applied in ecological studies in Norway^53,54^. We also applied modelled values of median surface salinity, as well as mean, maximum, and minimum surface seawater temperatures. These environmental parameters were retrieved from fjord-scale hydrodynamical models which are downscaled from the Norwegian coastal model, NorKyst800, to 160 m × 160 m horizontal resolution^55^. The fjord model system covers the entire Norwegian coast, separated into 13 model domains and applies the ocean model Regional Ocean Modeling System (ROMS, see http://myroms.org)^56,57^. Salinity and temperature values were extracted from the models corresponding to the core sampling sites by using the nearest grid cell. A similar model was validated for the Hardangerfjord on the west coast of Norway^58^ and another comparable hydrodynamic model system has been applied to predict kelp biomass^59^.

**Table 2:** Summary of environmental and eelgrass predictor variables used in this study and their relevance to carbon stocks and the key processes involved. See table S2 for the details of environmental variables in each site used in the analysis.

Variable name	Description and reference	Relevance to carbon stocks and key processes
Wave exposure	Modelled (25 × 25 m spatial resolution based on fetch and wind strength in 16 directions, averaged over a 10-year period 1995–2004)^60^	High wave exposure leads to more mobile sediments and lower particle trapping, as well as higher carbon loss from erosion and resuspension^50,61^
Water depth	Measured in situ	Deeper areas are less exposed to wave exposure (see above). Shallow areas, especially sheltered ones, are more likely to accumulate particles and detritus from waves as well as terrestrial runoff.
Salinity	Modelled (160 × 160 m spatial resolution, based on three years, May 2017-Apr 2020)^58^	Low salinity indicates that the site is located close to a river outlet, which further indicates inputs of organic matter from terrestrial and riverine sources^62,63^. As river outlets are often in sheltered areas, salinity is also correlated with wave exposure thus low salinity areas are more prone to accumulate organic matter.
Temperature (mean, minimum, and maximum)	Modelled (160 × 160 m spatial resolution based on daily values for three years, May 2017-Apr 2020)^58^	Temperature affects eelgrass and macroalgae survival, growth, and production, as well as decomposition rates of organic matter. Hence it affects carbon production as well as accumulation of carbon stocks. Note that latitude was highly correlated with temperature variables (R^2^ > 0.9) and so not included separately in the analysis.
Dry bulk density (DBD)	Calculated, described above	Related to wave exposure and sediment type: siltier, muddier sediments have lower DBD and are linked to low wave exposure values^36,37^
Eelgrass aboveground biomass	Dry weight: sampled, described above	Higher eelgrass biomass leads to higher detritus production, and higher particle trapping^64,65^
Eelgrass belowground biomass	Dry weight: sampled, described above	High belowground biomass enhances plant anchoring in exposed conditions, and is thus linked to higher wave exposure, reduced erosion, and increased sediment stability^66^
Eelgrass ag: bg ratio	Ratio of two above variables	Linked to hydrodynamics and light exposure^67^

### Statistical analysis

We used Generalized Linear Mixed Models (GLMM) to test whether carbon stocks and δ^13^C values differed significantly between samples taken from eelgrass meadows and those from unvegetated sediments. The analysis assumed a gamma distribution and included “Site” as a random factor, using the *lme4* package in R version 4.3.0^68^. In this analysis, we only included the Skagerrak sites (Sømskilen, Ærøya, Merdø, Langerompa, and Hove), and treated the two water depths at Langerompa as separate “sites”.

We used partial least squares (PLS) regression analysis, using the R-package *pls*^69^ to determine the importance of predictor variables on carbon stocks in both eelgrass and unvegetated sediments. PLS regression analysis has often been used in studies of seagrass carbon stocks^16,17,70^ due to its ability to cope with collinearity between predictor variables^71^. We checked the collinearity between environmental variables, and identified only one potentially notable correlation: between mean temperature and maximum temperature, (R2 = 0.91 and 0.84 in eelgrass and unvegetated sites, respectively. All other correlations were below 0.8 (Figure S1). Eelgrass and unvegetated samples were analysed separately because of the lack of biomass co-variables for unvegetated samples. The following predictor variables were included in the model selection procedure: water depth, wave exposure, salinity, mean water temperature, maximum water temperature, minimum water temperature, DBD, eelgrass aboveground biomass, eelgrass belowground biomass, and the ratio of eelgrass aboveground to belowground biomass (the last three variables were included in the analysis of eelgrass meadows only).

### Extrapolations, projections, and global comparisons

We extrapolated our results to projected carbon stocks for the top one meter of sediment per area, expressed in Mg C ha^−1^, to enable a rough comparison with other eelgrass meadows and to other blue carbon habitat, as other large-scale comparative studies have done^5,16^. To compare carbon stocks in eelgrass (*Zostera* spp.) sediments, we conducted a comprehensive literature search of peer-reviewed publications (see Appendix 1 for search terms and detailed methodology), resulting in 385 data points from 54 publications. These studies varied widely in terms of sampled sediment depth, ranging from 5 to 200 cm, as well as multiple different units of carbon density (per area and/or volume). To ensure consistence and allow for comparisons, we standardized all values to Mg C ha^−1^ for the top meter of sediment. Previous studies of seagrass meadows have shown that measuring carbon stocks in the top 25–50 cm provides a reliable estimate of carbon stocks within the top meter of sediment^5,72^. However, sites with low sediment accumulation rates will tend to be overestimated, whereas sites with high sediment accumulation can be underestimated, and this comparison should be considered with those caveats in mind. We also use the results from the sediment profiles in the 20–110 cm and 60–110 cm sections of the two deep cores to further comment on this assumption and its implications for estimating carbon stocks at a national scale.

## Results

### Sediment carbon stocks

**Fig. 2:**
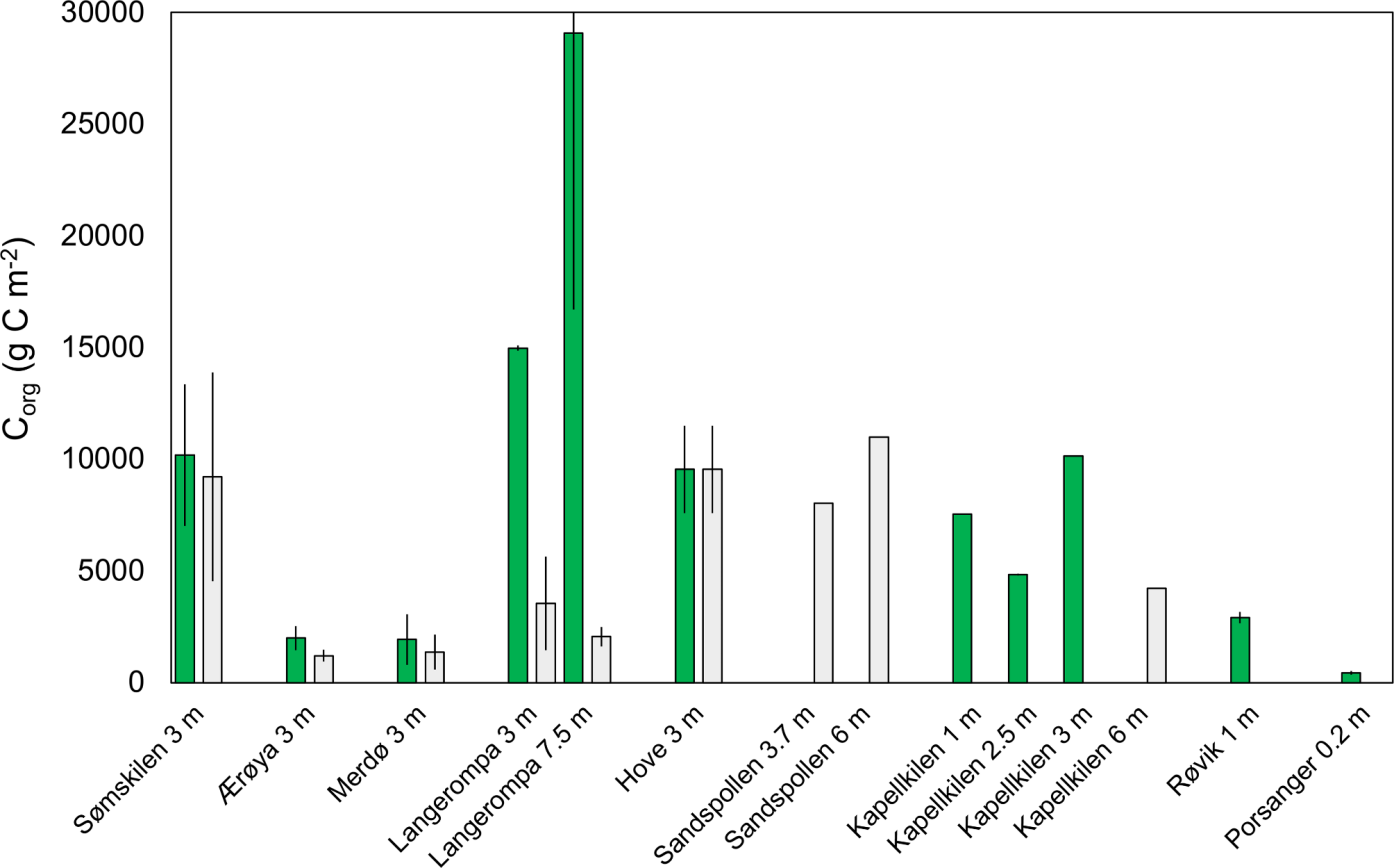
Sediment organic carbon stocks estimated for the top 50 cm of sediment (g C m^−2^, mean ± SE, *n* = 1–4) for both eelgrass (dark green) and nearby unvegetated (light grey) sediments. Sites are arranged geographically from south to north (i.e. left to right), with water depth given after each site name.

Sediment organic carbon stocks were highly variable across sites, with values ranging from 400 to 30 000 g C m^-2^ (Fig. 2). In all sites where both eelgrass and unvegetated sediments were sampled, carbon stocks were consistently higher in the eelgrass compared to unvegetated sediments within the same site. However, the magnitude of this difference was highly variable: carbon stocks in eelgrass and unvegetated sediments were very similar in Sømskilen and Hove, but substantially higher in eelgrass than unvegetated sediments in Langerompa (Fig. 2).

**Fig. 3:**
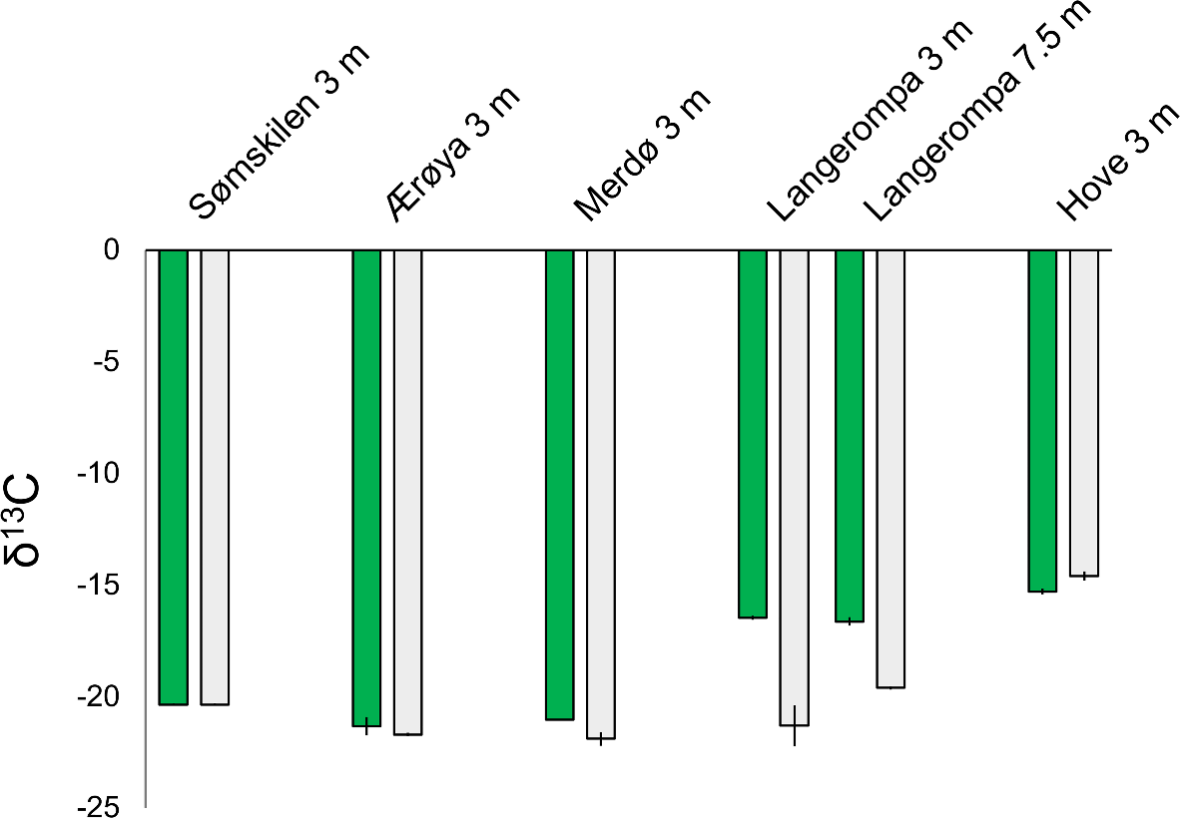
Sediment δ^13^C values of sediments (mean ± SE, *n* = 2–4) for both eelgrass (dark green) and nearby unvegetated (light grey) sediments in the Skagerrak sites.

δ^13^C was also higher in eelgrass compared to unvegetated sediments in all but one site (Fig. 3). Without sampling of the δ^13^C values of potential carbon sources, we could not precisely determine the respective contributions of eelgrass versus non-eelgrass carbon sources in the sediment. However, the elevated δ^13^C values observed, such as in Langerompa, may suggest higher contribution of eelgrass to sediment carbon than in unvegetated sediments, as eelgrass has higher δ^13^C values than macroalgal and riverine sources. As with carbon stocks, the highest difference between eelgrass and unvegetated sediment was in Langerompa (Fig. 3).

### Carbon depth profiles

**Fig. 4:**
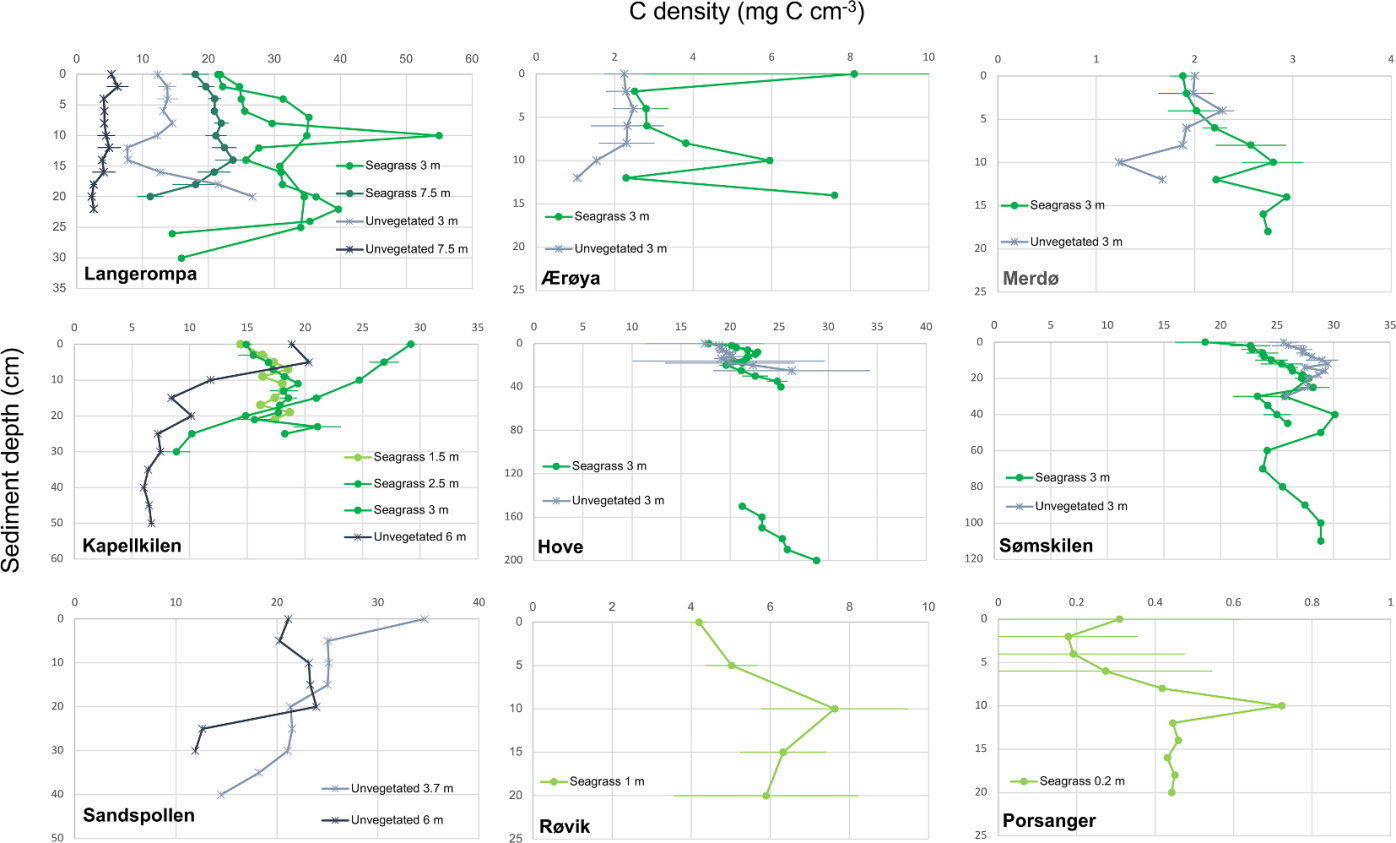
Sediment profiles of organic carbon density (mg C cm^−3^, mean ± SE) in relation to sediment depth (cm) for all nine study sites. Water depth and habitat are depicted as different symbols and colors, where green circles = eelgrass (light green: 0–1.5 m, medium green: 2.5–4 m, dark green: 6–7.5 m), grey crosses = unvegetated (light grey: 3–4 m, dark grey: 6–7.5 m). Two sediment profiles are shown at 3 m water depth in Langerompa as the two cores were sliced at different intervals and thus it was not possible to take the mean. Note the differing scales of x and y axes in different sites, and that the sediment depth denotes the length of the core, not necessarily how far the core penetrated within the sediment (see Table S1).

Despite high variation in the carbon density profiles across sites, some general patterns appeared (Fig. 4). At most sites, the eelgrass sediments had higher carbon densities compared to those from the unvegetated sediments. This pattern was evident in 3 out of 6 sites in the North Sea region (Langerompa, Ærøya and Kapellkilen). For the remaining sites within this region, this pattern is valid for some parts of the sediment profile: at Hove, it holds for the upper 20 cm, while at Merdø it holds from 6 cm and deeper. Conversely, in Sømskilen, the opposite pattern occurred, with higher carbon density values recorded in the upper 20 cm layer of the unvegetated sediment. The opposite pattern for Sømskilen could reflect that the currently unvegetated area was previously inhabited by eelgrass. For sites where sampling had been performed at several depths within eelgrass sediments, the highest carbon density values were observed at 3 m water depth, indicating that the carbon stock profile might reflect an optimal productivity depth within the meadows. Within the unvegetated sediments, the carbon density values either remained constant or decreased with sediment depth. One exception was Langerompa at 3 m water depth, where the values increased below 14 cm within the sediment, which could also reflect historical presence of eelgrass within this area. Also at Hove there seemed to be an increase in the carbon density in the deepest part of the profile in the unvegetated sediment, indicating historical presences of eelgrass or other carbon sources. The two deep cores at Hove and Sømskilen both showed higher carbon densities within deep (> 100 cm) sediments than in the upper layers (Fig. 4).

### Importance of environmental predictors

**Fig. 5:**
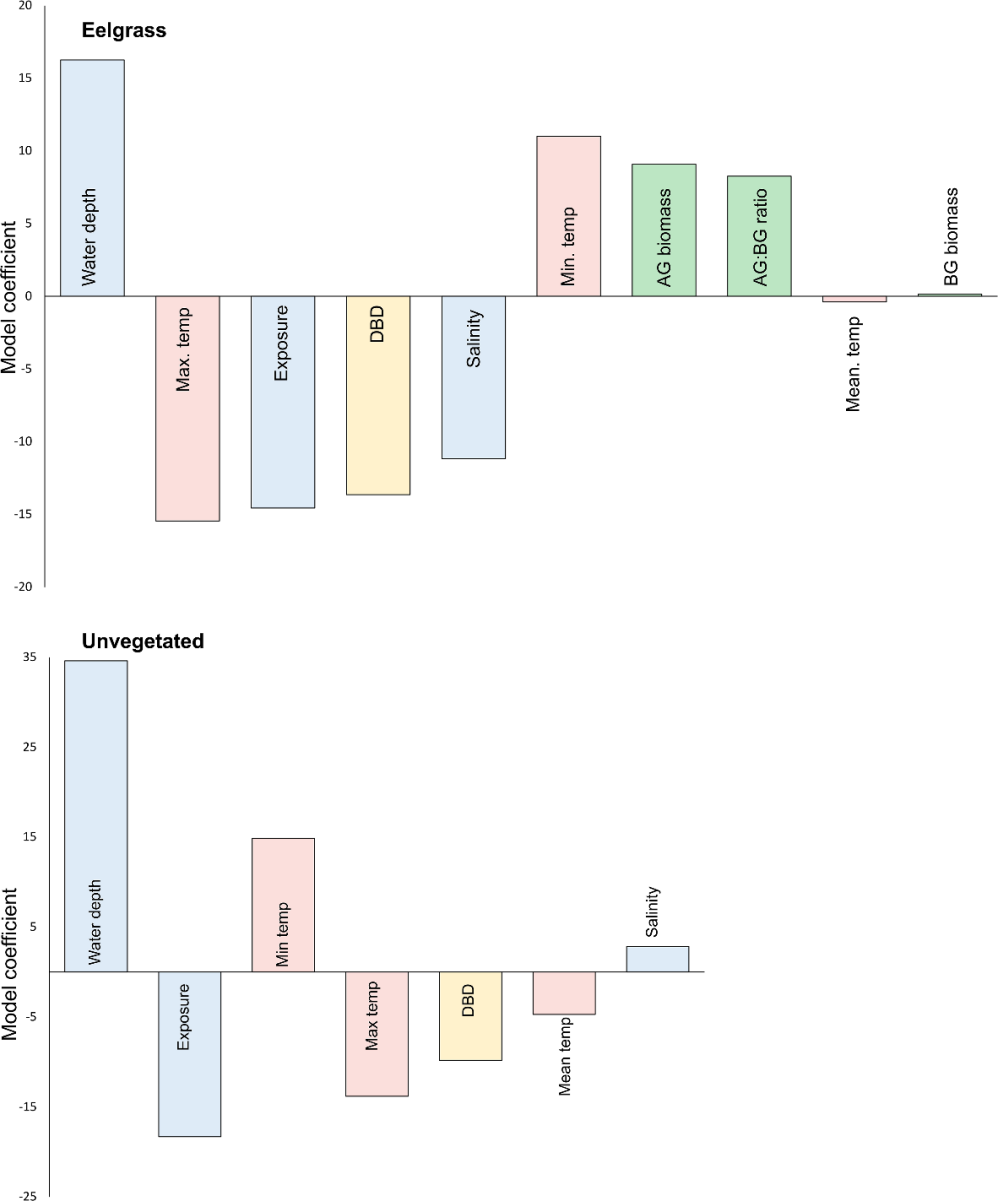
Coefficient plots of partial least squares regression models for sediment organic carbon stocks sampled from eelgrass (top panel) and unvegetated (bottom panel) samples, respectively. Predictor variables are standardised and ranked in order of importance from left to right, and the direction indicates the relationship with carbon stocks. Variables related to eelgrass are in green, sediment in yellow, temperature in red, and hydrodynamics/salinity in blue. BG biomass = belowground eelgrass biomass, AG biomass = aboveground eelgrass biomass, AG: BG ratio = aboveground to belowground eelgrass biomass ratio, DBD = sediment dry bulk density.

The importance of environmental predictor variables in explaining carbon stock values were overall similar in both eelgrass and unvegetated sediments (Fig. 5). In eelgrass meadows, water depth was the most important predictor variable, and positively correlated with carbon stocks. This was followed by maximum surface water temperature, wave exposure, DBD, and salinity, all of which were negatively correlated with carbon stocks. Minimum temperature, aboveground eelgrass biomass, and eelgrass aboveground: belowground ratio were also positively correlated with carbon stocks, while the correlations for mean temperature and eelgrass belowground biomass were close to zero (Fig. 5). In unvegetated sediments, high carbon stock values were correlated with water depth, as in the eelgrass meadows, followed by a negative correlation with wave exposure, positive correlation with mean temperature, and finally negative correlations with maximum temperature, DBD, and mean temperature (Fig. 5).

## Discussion

### Norwegian carbon stocks

The carbon stock values within eelgrass sediments were highly variable across the Norwegian coast ranging from 436 to 29 077 g m^−2^ (mean: 8 509 g m^−2)^ for the top 50 cm of sediment. Carbon stocks were also variable across regions with the highest stocks observed in the Skagerrak (11 290 g C m^−2)^ and Oslofjord (7 508 g C m^−2^) followed by the Norwegian Sea (2 905 g C m^−2^) and the lowest in the Barents Sea (43 g C m^−2^). Though this might imply higher carbon stocks in southern sites than northern ones, whether this truly reflects a latitudinal trend is uncertain as the latter three areas were represented by only one site each. In addition, within the Skagerrak, where multiple sites were sampled, we observed high variation in carbon stocks ranging from 1 991 to 29 077 g C m^−2^. Carbon stocks in unvegetated sediments were also variable and relatively high, ranging from 1 204 to 9 541 g C m^−2^ (though this only includes sites in the Skagerrak and Oslofjord) but systematically lower than in adjacent eelgrass sediments.

Based on the estimated Norwegian eelgrass areal coverage (90 km^2^)^40^, this represents a total sediment carbon stock in Norwegian eelgrass meadows ranging between 0.04 and 2.6 (mean: 0.77) Mt C. This is a first estimate of Norwegian eelgrass blue carbon stocks based on Norwegian data, though the values are within the same range as those estimated from a weighted average for the Nordic region multiplied by the Norwegian eelgrass coverage (0.25 (range: 0.06–0.4) Mt C for the top 25 cm^40^). The high variability in carbon stocks, as well as the potential variation caused by our approach of using data compiled from multiple sampling efforts and methodologies, and the different sediment depths used in the calculations across the literature (discussed below), makes it challenging to precisely assess the contribution of eelgrass blue carbon to national inventories. Nonetheless, both estimates align to support a national eelgrass carbon stock inventory within this range. To improve estimates, the uncertainty in the carbon stocks, as well as in the mapping and areal coverage of eelgrass meadow should be reduced. More accurate estimates would need a larger dataset of carbon stocks with multiple replicates across a wide range of environmental variables, such as a dedicated field sampling programme with a standardised sampling protocol to collect a representative set of sediment cores across the entire latitudinal and environmental range of Norwegian eelgrass meadows. Estimates of eelgrass distribution and cover could be improved by combining field surveys with novel drone and satellite remote sensing for large-scale systemic mapping efforts^73,74^. Further refining models on how the environmental factors discussed below affect carbon stocks could help develop predictive models for carbon stocks along the entire extensive Norwegian coastline, and eventually be adapted and applied to other regions.

### Environmental drivers of carbon stocks in eelgrass and unvegetated sediments

**Fig. 6:**
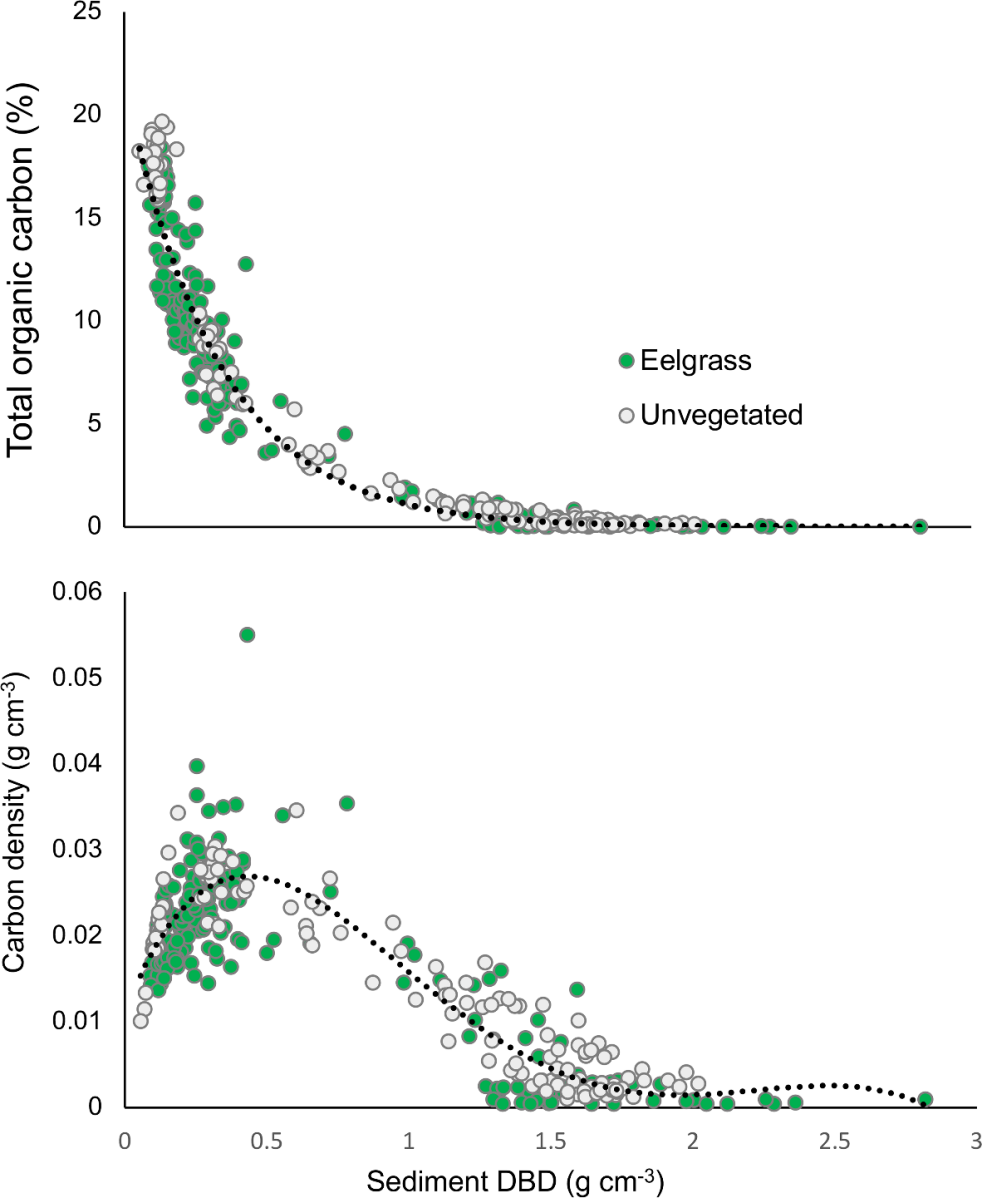
Relationship of sediment dry bulk density (DBD) with total organic carbon (top) and carbon density (bottom). Dark green points indicate data from eelgrass meadow sediments, and light grey points indicate data from unvegetated sediments. Fitted trendlines are negative exponential (top) and fifth-order polynomial (bottom).

These results, along with other studies, show that carbon stocks in seagrass meadows are highly variable. Among the drivers for this variation included in this study, water depth was the most important predictor variable of carbon stocks in eelgrass meadow sediments, though much of this was likely driven by extremely high carbon density values in the 7 m samples at Langerompa. Sediments in deeper water are less susceptible to hydrodynamic processes that disturb sediment and can cause a loss of sediment carbon^61^, potentially leading to higher carbon accumulation than shallower areas. However, most of our other samples were taken in the 2–3 m depth zone, so more intensive sampling across a wider depth range would be needed to truly verify the role of depth in determining sediment carbon stocks. Supporting the importance of hydrodynamics in driving carbon stocks, wave exposure was also strongly negatively correlated with sediment carbon, as high wave exposure rather promotes sediment resuspension and carbon loss rather than retention and sequestration^61,75^. Wave exposure is also highly linked to sediment characteristics, which many previous studies have identified as important predictors of seagrass carbon stocks on different spatial scales^16,17,19,50,76^. Hence, wave exposure can often be used a proxy of sediment characteristics. Here, we included dry bulk density (DBD) as our sole sediment characteristic, as we did not measure grain size or porosity. DBD is generally correlated with sediment grain size and type (low DBD being associated with small grain size and higher mud/silt content), and we indeed found it to be an important variable in our analysis, and negatively correlated with carbon stocks, indicating higher carbon content in muddier sites (e.g. Sømskilen, Hove, Langerompa; Table 1).This observation was supported by a strong negative and non-linear relationship between DBD and sediment carbon stocks in both eelgrass and unvegetated sediments, with the highest carbon densities found at DBD of 0.5 g cm^−3^ (Fig. 6).

In addition to hydrodynamic and sediment variables, both water temperature and salinity were identified as important drivers in our analysis. In particular, carbon stocks were negatively correlated with salinity (i.e. higher near river inlets). However, this is likely not a direct effect of salinity on carbon stocks, but rather an indication that environmental conditions in low-salinity sites are more conducive to high carbon stocks. Proximity to river outlets, where salinity is low, likely contributes to higher carbon stocks due to the input of particulate organic matter from terrestrial and riverine sources, irrespective of the presence of eelgrass^51,72^. In many seagrass meadows, a large proportion of sediment carbon is derived from external sources^9,16^, and thus the characteristics of the surrounding marine and terrestrial systems (presence of macroalgae beds, phytoplankton abundance, land use, human disturbances) are likely drivers of the carbon stocks within seagrass meadows. Many of the low-salinity sites in this study, for example Sømskilen, which had one of the highest carbon stock values, are also relatively sheltered from oceanic waves and currents, which also promotes sediment retention and accumulation. Interestingly, minimum seawater temperature was positively correlated with carbon stocks, but maximum seawater temperature was negatively correlated. Temperature is known to affect carbon storage and carbon cycling, by e.g. driving decomposition rates and methane production^77,78^. However, all but two of our sites were in the Skagerrak and Oslofjord, with minimal differences in temperature between them, and thus the trend we observed may be driven by the inclusion of the two sites in the Norwegian and Barents Sea which were both much colder and had low carbon values. Due to the lack of previous studies on blue carbon stocks at high latitudes, it is also difficult to determine whether the low values at these sites were due to temperature, the shallower water depth (intertidal in the case of the Barents Sea site), or indirect effects such as lower runoff of organic matter from land. Without including more sites across the entire temperature gradient of the Norwegian coast, it is difficult to predict the future impacts of climate change on carbon stocks in eelgrass meadows, especially in combination with uncertainty on the impacts of climate change on other interacting processes that can affect blue carbon^79^ such as methane production^77,78,80^ and decomposition^81^, particularly in northern sites where climate change is more pronounced^82^.

Eelgrass biomass has also previously been noted as an important driver in sediment carbon stocks^16^. The aboveground components of eelgrass (shoots and leaves) promote sediment trapping^65^ thus likely increasing carbon deposition rates into the sediment, while the belowground components (roots and rhizomes) limit sediment erosion and resuspension^83^ likely increasing carbon retention rates. While we found positive associations between carbon stocks and both aboveground eelgrass biomass and aboveground: belowground ratio and carbon stocks, but these were less important than the abiotic variables. In addition to the physical structure of the living eelgrass plants, eelgrass material such as detached leaves and dead rhizomes can be an important component of sediment carbon stocks^9,16^. We noted slightly higher (i.e. less negative) δ^13^C values in sediments of eelgrass compared to unvegetated sediments, which might indeed show the incorporation of eelgrass-derived carbon (which generally show higher δ^13^C than macroalgae or plankton) in these sediments, but precisely determining carbon origin is impossible without measuring potential sources. However, we also found that sediment δ^13^C values were much lower than those generally reported for eelgrass, suggesting high contribution of external sources such as macroalgae, plankton, or, especially in sites near river outliers, riverine and terrestrial sources, to sediment carbon stocks in eelgrass meadows.

While carbon stocks in unvegetated areas were generally lower than in adjacent eelgrass meadows, sites with higher carbon stock values in eelgrass sediments also had higher carbon stock values in unvegetated sediments (e.g. Langerompa and Hove). Given that the unvegetated areas were quite close to the meadows, this might reflect an overspill effect of the trapping and retention capacity of eelgrass, where some of the particles are deposited in areas near the edge of the meadow^84^. However, it is also possible that these high carbon stock values represent unvegetated areas which were previously vegetated, as evidenced by increasing carbon densities (and visual changes in sediment characteristics) in deeper sediment depths in these sites. It is also possible that the environmental characteristics of these sites (shallow, sheltered, muddy) with high carbon stocks in unvegetated sediments, promote the deposition of organic matter, which is even further enhanced by the presence of eelgrass, as evidenced by the higher stocks in eelgrass than unvegetated sediments. Similar carbon stock values in eelgrass and unvegetated sites were recently reported for Danish coastal waters^76^. This supports our findings that the presence of eelgrass itself is not the sole driver of sediment carbon stocks, and environmental drivers and sediment characteristic may be of similar importance.

### The importance of considering sediment depth and differing methodologies

In the global comparison below, we standardised carbon stocks from a variety of sediment depths (from 5 to 200 cm) to enable comparison across different studies. Extrapolated estimates of carbon stocks across depths are commonly used^5,72^, based on the assumption that carbon density is equally distributed across the sediment depth. However, both here and in other studies^8^, vertical profiles of sediment density in eelgrass meadows can be highly variable and can indicate different remineralisation rates, different bioturbation regimes, and/or changes in organic matter availability and accumulation over time. In most of our sites, organic carbon density increased with sediment depth, and this was also the case for the two deep cores (in Hove and Sømskilen). This might indicate a decrease in organic matter inputs in more recent years^8^, or an artifact of sampling methodology due to higher compaction in the lower core, as mentioned in the Methods section. Sediment dating or repeated sampling would be necessary to verify changes in sequestration rates, while methodological bias could be examined using replicated samplings at a range of penetration depths using open-barrel cores or alternate samplers allowing access to the entire length of the sediment core to retrieve undisturbed subsamples in the field.

Though only a few studies have explored carbon stocks below 100 cm depth, several have noted an increase in carbon density with depth in eelgrass meadows^63,85,86^) though not necessarily in unvegetated areas^86^. It is unclear how much sediment compression during the coring process affects these values, but it is likely that extrapolating total carbon content from shallow layers likely does not give a full picture of carbon stocks. In cases where total sediment depth is very shallow (i.e. exposed sites on bedrock or glacial clay) these extrapolations may be highly overestimated, while in sites with very deep soft sediments these are likely underestimated. In some of our sites (e.g. Langerompa, Sømskilen, Ærøya) we noted sudden changes in carbon density, which likely represent abrupt changes in sediment composition such as historical changes in vegetation cover (i.e. loss or shifts in eelgrass extent). We thus note that assuming constant carbon values over sediment depth is oversimplistic and likely to lead to erroneous carbon stock estimates.

### Eelgrass carbon stocks in a global context

**Fig. 7:**
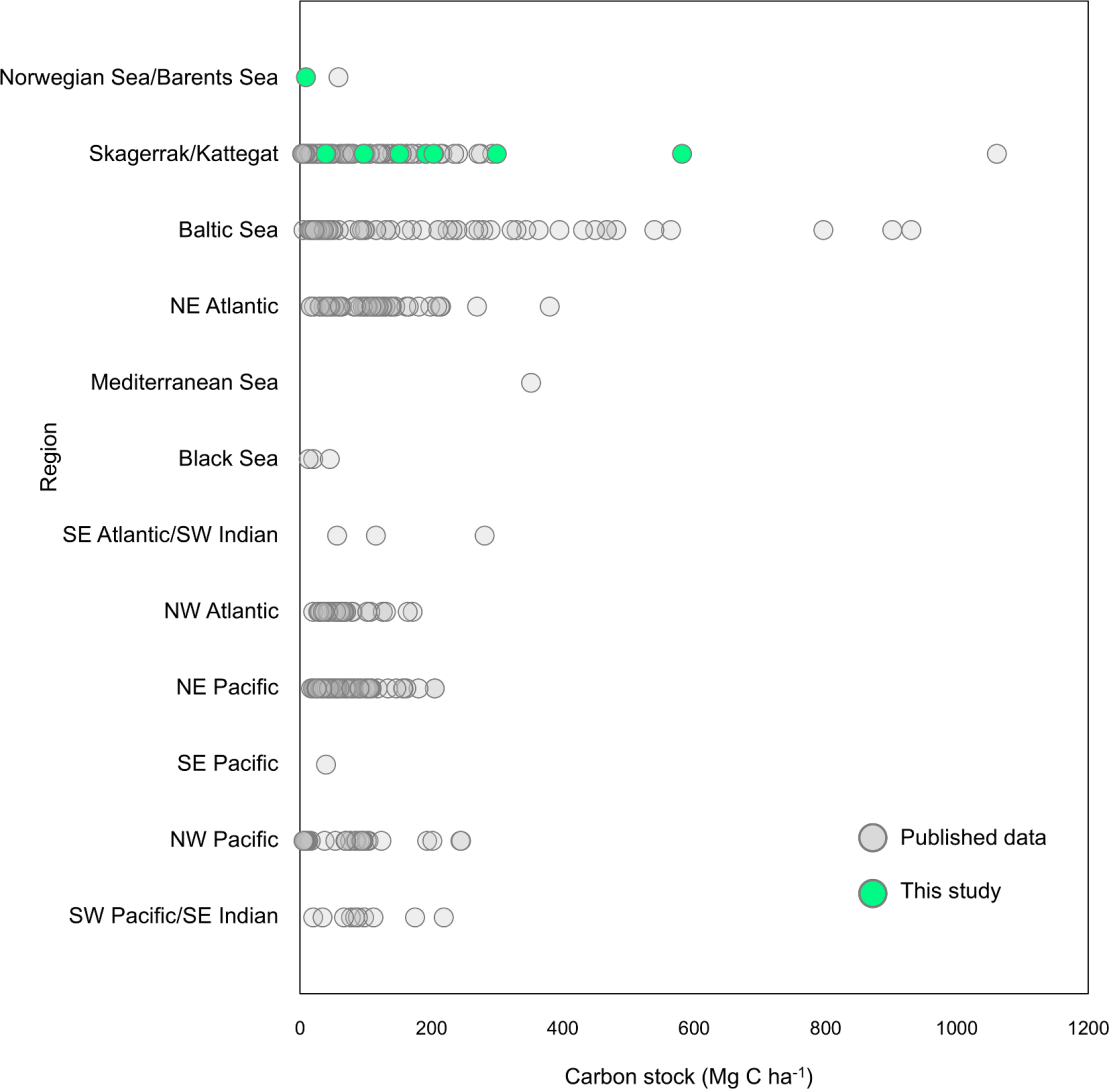
Estimated carbon stocks in the top 1 m of sediment (Mg C ha^−1^) in eelgrass (*Zostera* spp.) meadows in different global regions. Data points in grey are from published literature, while points in green are new data from this study (note that one point in grey in the Norwegian-Barents Sea region represents Røvik, which was previously published but also used in the analyses of this study). Studies use different methods, sediment depths, and units for calculating carbon stocks, therefore all data was converted to equivalent units for comparison, though see text for caveats of this approach. See Appendix 1 for full details and citations.

Within comparative studies at a global scale, carbon stocks are often given for the top meter of sediment^5,87^ especially to enable cross-ecosystem comparisons to other coastal or terrestrial systems. After converting our values to enable such comparisons, the carbon stocks measured in this study fit into the upper range of established northeastern Atlantic eelgrass carbon stocks (Fig. 7), even though our highest stocks were lower than those found in muddy Danish and German meadows in the Kattegat and western Baltic Sea^16,18,88^. Our results reinforce the idea of the NE Atlantic (Skagerrak, Kattegat, Baltic areas) as a region supporting sites with some of the highest eelgrass carbon stocks globally, with high conservation relevance^89^ due to the historical loss of many eelgrass meadows in this area^90,91^ and recent efforts at large-scale restoration^92,93^. In a global seagrass context, these values are higher than mean seagrass carbon stocks^5^, but still lower than the highest values recorded for *Posidonia oceanica* meadows^94^. Across ecosystems, eelgrass carbon stocks measured are, on a areal basis, similar to those found in tropical and temperate forests, and salt marshes, though lower than those in boreal forests and mangroves^16,95–99^. However, as discussed above, though useful, such comparisons based on extrapolation of data across sediment depths should be considered as highly uncertain. Proper comparison should consider the actual sediment accumulation and vertical profiles of carbon densities rather than simple linear extrapolation.

### Uncertainty, knowledge gaps, and management

We have shown here that assessing carbon stocks with high precision requires a better understanding of the variation across environmental gradients, as well as consideration of different sampling methods used. Despite high variability, our study further demonstrates the magnitude of eelgrass sediment carbon stocks, and supports the importance of preserving eelgrass meadows to ensure these blue carbon stocks are not released following eelgrass loss^91^. Conservation and restoration projects have been effective in improving the status of seagrass ecosystems in several regions and consequently in enhancing seagrass carbon stocks^100^. While conserving should be considered the most effective measure to prevent the release of accumulated sediment carbon, carbon stocks can also begin accumulating relatively quickly following restoration in areas where eelgrass has been lost^6,101,102^.

Understanding environmental drivers in order to provide accurate estimates of seagrass carbon stocks is also essential in assessing the effectiveness of both conservation and restoration efforts and in planning future projects. For example, our results underline the importance of sediment characteristics and wave exposure in driving carbon stocks in northern temperate eelgrass meadows. Here, prioritising the conservation and restoration of eelgrass meadows in muddy, sheltered areas could ensure the protection of blue carbon habitats, thus maximizing carbon sequestration into the sediments, reducing loss of carbon stocks, and promoting the recovery of lost stocks. The importance of these sites is further underlined by the fact that coastal sheltered areas are often the most affected by human impacts such as eutrophication, coastal development, construction, and dredging. These human impacts can affect the carbon stocks in seagrass meadows directly, through the degradation and loss of meadow area which leads to erosion and release of sediment carbon^51^, as well as indirectly. For example, the carbon sink capacity of shallow estuarine seagrasses is known to be weakened by eutrophication and coastal development^103^.

However, a holistic approach to conservation should aim at protecting a diversity of eelgrass meadows which contribute to multiple ecosystem services^104^, not only blue carbon. Eelgrass meadows in exposed areas may have lower carbon stocks, but likely host diverse communities which differ from those in sheltered areas. Preventing loss of populations and preserving intraspecific diversity is also important for species adaptive capacity in a changing environment^105,106^. Here, for the first time, we sampled the carbon stocks of one of the northernmost eelgrass populations in the Barents Sea. Though the carbon stock at this site was low, these northern eelgrass populations will experience the highest magnitude of global change in the coming years, and merit fruther study^107^. Understanding how future environmental conditions will affect carbon stocks both in relatively understudied and well-known meadows should be a priority for future blue carbon research^2^.

## Electronic supplementary material

Below is the link to the electronic supplementary material.

Supplementary Material 1

Supplementary Material 2

## Data Availability

All data generated or analysed in this study are included in the published article and its Supplementary Information files.

## References

[1] Herr, D. et al. Coastal ‘blue’ carbon: A revised guide to supporting coastal wetland programs and projects using climate finance and other financial mechanisms. https://portals.iucn.org/library/sites/library/files/documents/2015-066.pdf. 10.2305/IUCN.CH.2015.10.en (2016)

[2] Macreadie, P. I. et al. The future of Blue Carbon science. *Nat. Commun.* **10**, 3998 (2019). 31488846 10.1038/s41467-019-11693-w PMC6728345

[3] Duarte, C. M., Middelburg, J. J. & Caraco, N. Major role of marine vegetation on the oceanic carbon cycle. *Biogeosciences*. **2**, 1–8 (2005).

[4] Mcleod, E. et al. A blueprint for blue carbon: Toward an improved understanding of the role of vegetated coastal habitats in sequestering CO_2_. *Front*. *Ecol. Environ.* **9**, 552–560 (2011).

[5] Fourqurean, J. W. et al. Seagrass ecosystems as a globally significant carbon stock. *Nat. Geosci.* **5**, 505–509 (2012).

[6] Greiner, J. T., Wilkinson, G. M., McGlathery, K. J. & Emery, K. A. Sources of sediment carbon sequestered in restored seagrass meadows. *Mar. Ecol. Prog Ser.* **551**, 95–105 (2016).

[7] Mazarrasa, I. et al. Dynamics of carbon sources supporting burial in seagrass sediments under increasing anthropogenic pressure. *Limnol. Oceanogr.* **62**, 1451–1465 (2017).

[8] Kindeberg, T., Röhr, E., Moksnes, P. O., Boström, C. & Holmer, M. Variation of carbon contents in eelgrass (*Zostera marina*) sediments implied from depth profiles. *Biol. Lett.* **15**, 20180831 (2019). 31238855 10.1098/rsbl.2018.0831 PMC6597507

[9] Prentice, C. et al. A synthesis of blue carbon stocks, sources, and accumulation rates in eelgrass (*Zostera marina*) meadows in the northeast Pacific. *Glob. Biogeochem. Cycles*. **34**, (2020).

[10] Stankovic, M. et al. Quantification of blue carbon in seagrass ecosystems of Southeast Asia and their potential for climate change mitigation. *Sci. Total Environ.* **783**, 146858 (2021). 34088119 10.1016/j.scitotenv.2021.146858

[11] Fonseca, M. S. & Cahalan, J. A. A preliminary evaluation of wave attenuation by four species of seagrass. *Estuar. Coast. Shelf Sci.* **35**, 565–576 (1992).

[12] Miyajima, T., Koike, I., Yamano, H. & Iizumi, H. Accumulation and transport of seagrass-derived organic matter in reef flat sediment of Green Island, Great Barrier reef. *Mar. Ecol. Prog. Ser.* **175**, 251–259 (1998).

[13] Agawin, N. S. R. & Duarte, C. M. Evidence of direct particle trapping by a tropical seagrass meadow. *Estuaries*. **25**, 1205–1209 (2002).

[14] Gacia, E., Duarte, C. M. & Middelburg, J. J. Carbon and nutrient deposition in a Mediterranean seagrass (*Posidonia oceanica*) meadow. *Limnol. Oceanogr.* **47**, 23–32 (2002).

[15] Serrano, O. et al. Can mud (silt and clay) concentration be used to predict soil organic carbon content within seagrass ecosystems? *Biogeosciences*. **13**, 4915–4926 (2016).

[16] Röhr, M. E. et al. Blue carbon storage capacity of temperate eelgrass (*Zostera marina*) meadows. *Glob. Biogeochem. Cycles*. **32**, 1457–1475 (2018).

[17] Dahl, M. et al. Sediment properties as important predictors of carbon storage in *Zostera marina* meadows: A comparison of four European areas. *PLoS ONE*. **11**, e0167493 (2016). 27936111 10.1371/journal.pone.0167493 PMC5147920

[18] Röhr, M. E., Boström, C., Canal-Vergés, P. & Holmer, M. Blue carbon stocks in Baltic Sea eelgrass (*Zostera marina*) meadows. *Biogeosciences*. **13**, 6139–6153 (2016).

[19] Lima, M. D. A. C., Ward, R. D. & Joyce, C. B. Environmental drivers of sediment carbon storage in temperate seagrass meadows. *Hydrobiologia*. **847**, 1773–1792 (2020).

[20] McHenry, J. et al. Geographic variation in organic carbon storage by seagrass beds. *Limnol. Oceanogr.* **68**, 1256–1268 (2023).

[21] McKenzie, L. J. et al. The global distribution of seagrass meadows. *Environ. Res. Lett.* **15**, 074041 (2020).

[22] Macreadie, P. I. et al. Blue carbon as a natural climate solution. *Nat. Rev. Earth Environ.* **2**, 826–839 (2021).

[23] Liu, S. et al. Nutrient loading weakens seagrass blue carbon potential by stimulating seagrass detritus carbon emission. *Ecol. Ind.* **157**, 111251 (2023).

[24] Hughes, B. B. et al. Recovery of a top predator mediates negative eutrophic effects on seagrass. *Proc. Natl. Acad. Sci.* **110**, 15313–15318 (2013). 10.1073/pnas.1302805110 PMC3780875 23983266

[25] Nordlund, L. M. et al. One hundred priority questions for advancing seagrass conservation in Europe. *Plants People Planet.* **6**, 587–603. 10.1002/ppp3.10486 (2024).

[26] de los Santos, C. B. et al. Recent trend reversal for declining European seagrass meadows. *Nat. Commun.* **10**, 3356 (2019). 31350407 10.1038/s41467-019-11340-4 PMC6659699

[27] Dunic, J. C., Brown, C. J., Connolly, R. M., Turschwell, M. P. & Côté, I. M. Long-term declines and recovery of meadow area across the world’s seagrass bioregions. *Glob. Change Biol.* **27**, 4096–4109 (2021). 10.1111/gcb.15684 33993580

[28] Lovelock, C. E. & Duarte, C. M. Dimensions of blue carbon and emerging perspectives. *Biol. Lett.* **15**, 20180781 (2019). 30836882 10.1098/rsbl.2018.0781 PMC6451379

[29] De Duarte, M. & Macreadie, P. I. The evolution of blue carbon science. *Wetlands*. **42**, 109 (2022).

[30] Khan, M., Northrop, E. & Schindler Murray, L. *Ocean-Based Climate Action in New and Updated Nationally Determined Contributions* (World Resources Institute, 2022).

[31] Valckenaere, J., Techera, E., Filbee-Dexter, K. & Wernberg, T. Unseen and unheard: The invisibility of kelp forests in international environmental governance. *Front. Mar. Sci.* **10**, 1235952 (2023).

[32] Schindler Murray, L. et al. The blue carbon handbook: Blue carbon as a nature-based solution for climate action and sustainable development. (2023).

[33] Krause-Jensen, D. et al. Nordic blue carbon ecosystems: Status and outlook. *Front. Mar. Sci.* **9**, 847544 (2022).

[34] Moore, K. A. & Short, F. T. *Zostera* Biology, Ecology, and Management. In *Seagrasses: Biology, Ecology, and Conservation* 361–386 (Springer, 2006).

[35] Boström, C. et al. Distribution, structure and function of nordic eelgrass (*Zostera marina*) ecosystems: Implications for coastal management and conservation. *Aquat. Conserv. Mar. Freshw. Ecosyst.* **24**, 410–434 (2014). 10.1002/aqc.2424 PMC4497458 26167100

[36] Santos, R. et al. Superficial sedimentary stocks and sources of carbon and nitrogen in coastal vegetated assemblages along a flow gradient. *Sci. Rep.* **9**, 610 (2019). 30679706 10.1038/s41598-018-37031-6 PMC6345834

[37] Kindeberg, T., Ørberg, S. B., Röhr, M. E., Holmer, M. & Krause-Jensen, D. Sediment stocks of carbon, nitrogen, and phosphorus in Danish eelgrass meadows. *Front. Mar. Sci.* **5**, 474 (2018).

[38] Novak, A. B. et al. Factors influencing carbon stocks and accumulation rates in eelgrass meadows across New England, USA. *Estuar. Coast*. **43**, 2076–2091 (2020). PMC7751660 33364916

[39] Billman, M., Santos, I. R. & Jahnke, M. Small carbon stocks in sediments of Baltic Sea eelgrass meadows. *Front. Mar. Sci.* **10**, 1219708 (2023).

[40] Frigstad, H. et al. Blue carbon – climate adaptation, CO_2_ uptake and sequestration of carbon in Nordic blue forests. (2021). TemaNord2020 541.

[41] Norwegian Environment Agency. Greenhouse Gas Emissions 1990–2021. National Inventory Report. (2023).

[42] Potouroglou, M. *Assessing the role of Intertidal Seagrasses as Coastal Carbon Sinks in Scotland* (Phd Thesis, Edinburgh Napier University, 2017).

[43] Craig, H. Isotopic standards for carbon and oxygen and correction factors for mass-spectrometric analysis of carbon dioxide. *Geochim. Cosmochim. Acta*. **12**, 133–149 (1957).

[44] Wiedemeyer, W. L. & Schwamborn, R. Detritus derived from eelgrass and macroalgae as potential carbon source for *Mytilus edulis* in Kiel Fjord, Germany: A preliminary carbon isotopic study. *Helgolander Meeresun*. **50**, 409–413 (1996).

[45] Ha, S., Min, W. K., Kim, D. S. & Shin, K. H. Trophic importance of meiofauna to polychaetes in a seagrass (*Zostera marina*) bed as traced by stable isotopes. *J. Mar. Biol. Assoc. UK*. **94**, 121–127 (2014).

[46] Thormar, J. et al. Eelgrass (*Zostera marina*) food web structure in different environmental settings. *PLoS ONE*. **11**, e0146479 (2016). 26752412 10.1371/journal.pone.0146479 PMC4708997

[47] Gagnon, K. et al. Role of food web interactions in promoting resilience to nutrient enrichment in a brackish water eelgrass (*Zostera marina*) ecosystem. *Limnol. Oceanogr.* **66**, 2810–2826 (2021).

[48] Olson, A. M. et al. Grazing preference and isotopic contributions of kelp to *Zostera marina* mesograzers. *Front. Plant. Sci.* **13**, 991744 (2022). 36311148 10.3389/fpls.2022.991744 PMC9608150

[49] Glew, J. R., Smol, J. P. & Last, W. M. Sediment core collection and extrusion. In *Tracking Environmental Change Using lake Sediments, Developments in Paleoenvironmental Research* vol. 1 73–105 (*Springer*, 2001).

[50] Dahl, M. et al. The influence of hydrodynamic exposure on carbon storage and nutrient retention in eelgrass (*Zostera marina* L.) meadows on the Swedish skagerrak coast. *Sci. Rep.* **10**, 13666 (2020). 32788660 10.1038/s41598-020-70403-5 PMC7423977

[51] Dahl, M. et al. First assessment of seagrass carbon accumulation rates in Sweden: A field study from a fjord system at the Skagerrak coast. *PLoS Clim.* **2**, e0000099 (2023).

[52] Dahl, M. et al. High seasonal variability in sediment carbon stocks of cold-temperate seagrass meadows. *J. Geophys. Res. Biogeosci*. **125**, e2019JG005430 (2020).

[53] Bekkby, T. et al. The abundance of kelp is modified by the combined impact of depth, waves and currents. *Front. Mar. Sci.* **6**, 475 (2019).

[54] Rinde, E. et al. The influence of physical factors on kelp and sea urchin distribution in previously and still grazed areas in the NE Atlantic. *PLoS ONE* **9**, e100222 (2014). 10.1371/journal.pone.0100222 PMC4064999 24949954

[55] Asplin, L., Albretsen, J., Johnsen, I. A. & Sandvik, A. D. The hydrodynamic foundation for salmon lice dispersion modeling along the Norwegian coast. *Ocean. Dyn.* **70**, 1151–1167 (2020).

[56] Haidvogel, D. B. et al. Ocean forecasting in terrain-following coordinates: Formulation and skill assessment of the Regional Ocean modeling system. *J. Comput. Phys.* **227**, 3595–3624 (2008).

[57] Shchepetkin, A. F. & McWilliams, J. C. The regional oceanic modeling system (ROMS): A split-explicit, free-surface, topography-following-coordinate oceanic model. *Ocean. Model.* **9**, 347–404 (2005).

[58] Dalsøren, S. B., Albretsen, J. & Asplin, L. New validation method for hydrodynamic fjord models applied in the Hardangerfjord, Norway. *Estuar. Coast. Shelf Sci.* **246**, 107028 (2020).

[59] van Son, T. C. et al. Achieving reliable estimates of the spatial distribution of kelp biomass. *Front. Mar. Sci.* **7**, 107 (2020).

[60] Isæus, M., Malm, T., Persson, S. & Svensson, A. Effects of filamentous algae and sediment on recruitment and survival of *Fucus serratus* (Phaeophyceae) juveniles in the eutrophic Baltic Sea. *Eur. J. Phycol.* **39**, 301–307 (2004).

[61] Egea, L. G., Infantes, E. & Jiménez-Ramos, R. Loss of POC and DOC on seagrass sediments by hydrodynamics. *Sci. Total Environ.* **901**, 165976 (2023). 37536591 10.1016/j.scitotenv.2023.165976

[62] De Falco, G., Baroli, M., Murru, E., Piergallini, G. & Cancemi, G. Sediment analysis evidences two different depositional phenomena influencing seagrass distribution in the Gulf of Oristano (Sardinia, Western Mediterranean). *J. Coast Res.* **225**, 1043–1050 (2006).

[63] Miyajima, T. et al. Geographic variability in organic carbon stock and accumulation rate in sediments of East and Southeast Asian seagrass meadows. *Glob. Biogeochem. Cycles*. **29**, 397–415 (2015).

[64] Ward, L. G., Kemp, M., Boynton, W. R. & W. & The influence of waves and seagrass communities on suspended particulates in an estuarine embayment. *Mar. Geol.* **59**, 85–103 (1984).

[65] van Katwijk, M. M., Bos, A. R., Hermus, D. C. R. & Suykerbuyk, W. Sediment modification by seagrass beds: Muddification and sandification induced by plant cover and environmental conditions. *Estuar. Coast. Shelf Sci.* **89**, 175–181 (2010).

[66] Fonseca, M. S. & Bell, S. S. Influence of physical setting on seagrass landscapes near Beaufort, North Carolina, USA. *Mar. Ecol. Prog. Ser.* **171**, 109 (1998).

[67] Peralta, G., Brun, F., Pérez-Lloréns, J. & Bouma, T. Direct effects of current velocity on the growth, morphometry and architecture of seagrasses: A case study on *Zostera noltii*. *Mar. Ecol. Prog. Ser.* **327**, 135–142 (2006).

[68] Bates, D., Mächler, M., Bolker, B. & Walker, S. Fitting linear mixed-effects models using lme4. *J. Stat. Softw.* **67**, 1–48 (2015).

[69] Liland, K., Mevik, B. & Wehrens, R. Partial Least Squares and Principal Component Regression. R package version 2.8-2. (2023).

[70] Gullström, M. et al. Blue carbon storage in tropical seagrass meadows relates to carbonate stock dynamics, plant–sediment processes, and landscape context: Insights from the western Indian Ocean. *Ecosystems*. **21**, 551–566 (2018).

[71] Carrascal, L. M., Galván, I. & Gordo, O. Partial least squares regression as an alternative to current regression methods used in ecology. *Oikos*. **118**, 681–690 (2009).

[72] Lavery, P. S., Mateo, M. Á., Serrano, O. & Rozaimi, M. Variability in the carbon storage of seagrass habitats and its implications for global estimates of blue carbon ecosystem service. *PLoS ONE*. **8**, e73748 (2013). 24040052 10.1371/journal.pone.0073748 PMC3764034

[73] Borja, A. et al. Innovative and practical tools for monitoring and assessing biodiversity status and impacts of multiple human pressures in marine systems. *Environ. Monit. Assess.* **196**, 694 (2024). 38963575 10.1007/s10661-024-12861-2

[74] Kvile, K. Ø. et al. Drone and ground-truth data collection, image annotation and machine learning: A protocol for coastal habitat mapping and classification. *MethodsX.* **12**, 102935. 10.1016/j.mex.2024.102935 (2024). 10.1016/j.mex.2024.102935 PMC11409010 39295629

[75] Dahl, M. et al. Increased current flow enhances the risk of organic carbon loss from *Zostera marina* sediments: Insights from a flume experiment. *Limnol. Oceanogr.* **63**, 2793–2805 (2018).

[76] Leiva-Dueñas, C. et al. Capturing of organic carbon and nitrogen in eelgrass sediments of southern Scandinavia. *Limnol. Oceanogr.* **68**, 631–648 (2023).

[77] Burkholz, C., Garcias-Bonet, N. & Duarte, C. M. Warming enhances carbon dioxide and methane fluxes from Red Sea seagrass (*Halophila Stipulacea*) sediments. *Biogeosciences*. **17**, 1717–1730 (2020).

[78] George, R. *Seagrasses in Warming Oceans. Physiological and Biogeochemical Responses* (PhD Thesis, Stockholm University, 2019).

[79] Lovelock, C. E. & Reef, R. Variable impacts of climate change on blue carbon. *One Earth*. **3**, 195–211 (2020).

[80] Eyre, B. D., Camillini, N., Glud, R. N. & Rosentreter, J. A. The climate benefit of seagrass blue carbon is reduced by methane fluxes and enhanced by nitrous oxide fluxes. *Commun. Earth Environ.* **4**, 374 (2023).

[81] Ouyang, X. et al. Response of macrophyte litter decomposition in global blue carbon ecosystems to climate change. *Glob. Change Biol.* **29**, 3806–3820 (2023). 10.1111/gcb.16693 36946867

[82] Filbee-Dexter, K. et al. Kelp carbon sink potential decreases with warming due to accelerating decomposition. *PLoS Biol.* **20**, e3001702 (2022). 35925899 10.1371/journal.pbio.3001702 PMC9352061

[83] Marin-Diaz, B., Bouma, T. J. & Infantes, E. Role of eelgrass on bed‐load transport and sediment resuspension under oscillatory flow. *Limnol. Oceanogr.* **65**, 426–436 (2020).

[84] Navarrete-Fernández, T. et al. The role of seagrass meadows in the coastal trapping of litter. *Mar. Pollut Bull.* **174**, 113299 (2022). 35090282 10.1016/j.marpolbul.2021.113299

[85] Kauffman, J. B. et al. Total ecosystem carbon stocks of mangroves across broad global environmental and physical gradients. *Ecol. Monogr.* **90**, e01405 (2020).

[86] Watanabe, K., Seike, K., Kajihara, R., Montani, S. & Kuwae, T. Relative sea-level change regulates organic carbon accumulation in coastal habitats. *Glob Change Biol.* **25**, 1063–1077 (2019). 10.1111/gcb.14558 PMC6850580 30589156

[87] Fu, C. et al. Substantial blue carbon sequestration in the world’s largest seagrass meadow. *Commun. Earth Environ.* **4**, 474 (2023).

[88] Stevenson, A., Corcora, Ó., Hukriede, T. C., Schubert, W., Reusch, T. B. & P. R. & H. Substantial seagrass blue carbon pools in the southwestern Baltic Sea include relics of terrestrial peatlands. *Front. Mar. Sci.* **9**, 949101 (2022).

[89] Jahnke, M. et al. Potential and realized connectivity of the seagrass *Posidonia oceanica* and their implication for conservation. *Divers. Distrib.* **23**, 1423–1434 (2017).

[90] Moksnes, P. O., Eriander, L., Infantes, E. & Holmer, M. Local regime shifts prevent natural recovery and restoration of lost eelgrass beds along the Swedish west coast. *Estuar. Coast*. **41**, 1712–1713 (2018).

[91] Moksnes, P. et al. Major impacts and societal costs of seagrass loss on sediment carbon and nitrogen stocks. *Ecosphere*. **12**, e03658 (2021).

[92] Eriander, L. *Restoration and Management of Eelgrass (Zostera marina) on the west Coast of Sweden* (PhD Thesis, University of Gothenburg, 2016).

[93] Steinfurth, R. C. et al. Improved benthic fauna community parameters after large-scale eelgrass (*Zostera marina*) restoration in Horsens Fjord, Denmark*. Mar. Ecol. Prog. Ser.* **687**, 65–77 (2022).

[94] Serrano, O., Lavery, P. S., Rozaimi, M. & Mateo, M. Á. Influence of water depth on the carbon sequestration capacity of seagrasses. *Glob. Biogeochem. Cycles*. **28**, 950–961 (2014).

[95] Duarte, C. M., Losada, I. J., Hendriks, I. E., Mazarrasa, I. & Marbà, N. The role of coastal plant communities for climate change mitigation and adaptation. *Nat. Clim. Change*. **3**, 961–968 (2013).

[96] Siikamäki, J., Sanchirico, J. N., Jardine, S., McLaughlin, D. & Morris, D. Blue carbon: Coastal ecosystems, their carbon storage, and potential for reducing emissions. *Environ. Sci. Policy Sustain. Devel*. **55**, 14–29 (2013).

[97] Pendleton, L. et al. Estimating global blue carbon emissions from conversion and degradation of vegetated coastal ecosystems. *PLoS ONE*. **7**, e43542 (2012). 22962585 10.1371/journal.pone.0043542 PMC3433453

[98] Duarte, C. M. & Chiscano, C. L. Seagrass biomass and production: A reassessment. *Aquat. Bot.* **65**, 159–174 (1999).

[99] Kennedy, H. et al. Seagrass sediments as a global carbon sink: Isotopic constraints. *Glob. Biogeochem. Cycles.* **24,** GB4034 (2010).

[100] Rattanachot, E., Stankovic, M., Aongsara, S. & Prathep, A. Ten years of conservation efforts enhance seagrass cover and carbon storage in Thailand. *Bot. Mar.* **61**, 441–451 (2018).

[101] Thorhaug, A., Poulos, H. M., López-Portillo, J., Ku, T. C. W. & Berlyn, G. P. Seagrass blue carbon dynamics in the Gulf of Mexico: Stocks, losses from anthropogenic disturbance, and gains through seagrass restoration. *Sci. Total Environ.* **605–606**, 626–636 (2017). 28672251 10.1016/j.scitotenv.2017.06.189

[102] Lange, T. et al. Large-scale eelgrass transplantation: A measure for carbon and nutrient sequestration in estuaries. *Mar. Ecol. Prog. Ser.* **685**, 97–109 (2022).

[103] Macreadie, P. I., Allen, K., Kelaher, B. P., Ralph, P. J. & Skilbeck, C. G. Paleoreconstruction of estuarine sediments reveal human-induced weakening of coastal carbon sinks. *Glob. Change Biol.* **18**, 891–901 (2012).

[104] Nordlund, L. M., Koch, E. W., Barbier, E. B. & Creed, J. C. Seagrass ecosystem services and their variability across genera and geographical regions. *PLoS ONE* **11**, e0163091 (2016). 10.1371/journal.pone.0163091 PMC5061329 27732600

[105] Ehlers, A., Worm, B. & Reusch, T. B. H. Importance of genetic diversity in eelgrass *Zostera marina* for its resilience to global warming. *Mar. Ecol. Prog Ser.* **355**, 1–7 (2008).

[106] Norderhaug, K. M. et al. The International Union for Conservation of Nature Red List does not account for intraspecific diversity. *ICES J. Mar. Sci. fsae*. **81**, 815-822 (2024).

[107] Krause-Jensen, D. et al. Imprint of climate change on pan-arctic marine vegetation. *Front. Mar. Sci.* **7**, 617324 (2020).

