# Supplementary material for: Carbon stocks in Norwegian eelgrass meadows across environmental gradients
Source: Sci Rep. 2024 Oct 24;14:25171. doi: 10.1038/s41598-024-74760-3 (PMC11502847; doi:10.1038/s41598-024-74760-3)
Supplement: Supplementary file 2 — Supplementary Material 2 [file 41598_2024_74760_MOESM2_ESM.docx]

Supplementary material for the manuscript: “*Carbon stocks in Norwegian eelgrass meadows across environmental gradients*” Gagnon et al. 2024

**List of supplementary material**

Table S1. Detailed information on corers, sediment penetration depth (i.e. the depth to which the core was pushed to), and compressed core length (the final length of the core when it was sliced in the laboratory).

Table S2. Environmental variables for each site. Note that for informative purposes mean dry bulk density (DBD) for each site is presented here, however the individual DBD of each core was used in the analysis. Temperature and salinity are surface values.

Figure S1. Correlation matrix of environmental variables used in the PLS analysis

Appendix 1. Methodology of literature review on eelgrass carbon stocks

**Table S1. Detailed information on corers, sediment penetration depth (i.e. the depth to which the core was pushed to), and compressed core length (the final length of the core when it was sliced in the laboratory).**

| **Core name** | **Site name** | **Year** | **Coordinates** | **Habitat** | **Water depth (m)** | **Corer diameter (mm)** | **Corer length (cm)** | **Penetration depth (cm)** | **Compressed core length (cm)** |
| --- | --- | --- | --- | --- | --- | --- | --- | --- | --- |
| Søm A | Sømskilen | 2017 | 58.404 N, 8.714 E | Eelgrass | 3 | 56 | 100 | 100 | 45 |
| Søm B | Sømskilen | 2017 | 58.404 N, 8.714 E | Eelgrass | 3 | 56 | 100 | 100 | 45 |
| Søm C | Sømskilen | 2017 | 58.404 N, 8.714 E | Eelgrass | 3 | 56 | 100 | 100 | 45 |
| Søm Ø110 | Sømskilen | 2017 | 58.404 N, 8.714 E | Eelgrass | 3 | 110 | 200 | 200 | 110 |
| Søm EC | Sømskilen | 2017 | 58.404 N, 8.714 E | Unvegetated | 3 | 56 | 50 | 90 | 35 |
| Søm FC | Sømskilen | 2017 | 58.404 N, 8.714 E | Unvegetated | 3 | 56 | 50 | 90 | 35 |
| S1 | Ærøya | 2017 | 58.417 N, 8.763 E | Eelgrass | 3 | 56 | 50 | 16 | 16 |
| S3 | Ærøya | 2017 | 58.417 N, 8.763 E | Eelgrass | 3 | 56 | 50 | 8 | 8 |
| Control 1 | Ærøya | 2017 | 58.417 N, 8.763 E | Unvegetated | 3 | 56 | 50 | 6 | 6 |
| Control 2 | Ærøya | 2017 | 58.417 N, 8.763 E | Unvegetated | 3 | 56 | 50 | 10 | 10 |
| Control 3 | Ærøya | 2017 | 58.417 N, 8.763 E | Unvegetated | 3 | 56 | 50 | 14 | 14 |
| S1 | Merdø | 2017 | 58.428 N, 8.808 E | Eelgrass | 3 | 56 | 50 | 16 | 16 |
| S3 | Merdø | 2017 | 58.428 N, 8.808 E | Eelgrass | 3 | 56 | 50 | 8 | 8 |
| C1 | Merdø | 2017 | 58.428 N, 8.808 E | Eelgrass | 3 | 56 | 50 | 6 | 6 |
| C2 | Merdø | 2017 | 58.428 N, 8.808 E | Unvegetated | 3 | 56 | 50 | 10 | 10 |
| C3 | Merdø | 2017 | 58.428 N, 8.808 E | Unvegetated | 3 | 56 | 50 | 14 | 14 |
| 3mA (L) | Langerompa | 2017 | 58.431 N, 8.801 E | Eelgrass | 3 | 56 | 100 | 80 | 28 |
| 3mB (L) | Langerompa | 2017 | 58.431 N, 8.801 E | Eelgrass | 3 | 56 | 100 | 70 | 35 |
| 3mC (L) | Langerompa | 2017 | 58.431 N, 8.801 E | Unvegetated | 3 | 52 | 25 | 6 | 6 |
| 3mD (L) | Langerompa | 2017 | 58.431 N, 8.801 E | Unvegetated | 3 | 52 | 25 | 9 | 6 |
| LSC-1 | Langerompa | 2017 | 58.431 N, 8.801 E | Unvegetated | 3 | 56 | 50 | 50 | 22 |
| LDS-1 | Langerompa | 2017 | 58.431 N, 8.801 E | Eelgrass | 7.5 | 56 | 50 | 45 | 22.5 |
| LDS-2 | Langerompa | 2017 | 58.431 N, 8.801 E | Eelgrass | 7.5 | 56 | 50 | 45 | 22.5 |
| LDS-3 | Langerompa | 2017 | 58.431 N, 8.801 E | Eelgrass | 7.5 | 56 | 50 | 45 | 22.5 |
| LDC-1 | Langerompa | 2017 | 58.431 N, 8.801 E | Unvegetated | 7.5 | 56 | 50 | 35 | 26 |
| LDC-2 | Langerompa | 2017 | 58.431 N, 8.801 E | Unvegetated | 7.5 | 56 | 50 | 40 | 22 |
| 3mA (H) | Hove | 2017 | 58.448 N, 8.818 E | Eelgrass | 3 | 56 | 100 | 100 | 41 |
| 3mB (H) | Hove | 2017 | 58.448 N, 8.818 E | Eelgrass | 3 | 56 | 100 | 100 | 37 |
| 3mC (H) | Hove | 2017 | 58.448 N, 8.818 E | Eelgrass | 3 | 56 | 100 | 100 | 32 |
| 3mD (H) | Hove | 2017 | 58.448 N, 8.818 E | Eelgrass | 3 | 56 | 50 | 50 | 20 |
| Hove Day4 | Hove | 2017 | 58.448 N, 8.818 E | Eelgrass | 3 | 110 | 200 | 200 | 110 |
| Control A | Hove | 2017 | 58.448 N, 8.818 E | Unvegetated | 3 | 56 | 50 | 50 | 28 |
| Control B | Hove | 2017 | 58.448 N, 8.818 E | Unvegetated | 3 | 56 | 50 | 50 | 30 |
| Sand 3.7 | Sandspollen | 2018 | 59.663 N, 10.590 E | Unvegetated | 3.7 | 110 | 100 | 68 | 45 |
| Sand 6.0 | Sandspollen | 2018 | 59.663 N, 10.590 E | Unvegetated | 6 | 110 | 100 | 37 | 31 |
| Kapell 3 | Kapellkilen | 2018 | 59.666 N, 10.587 E | Eelgrass | 3 | 110 | 100 | 41 | 34 |
| Kapell 6 | Kapellkilen | 2018 | 59.666 N, 10.587 E | Eelgrass | 6 | 110 | 100 | 63 | 52 |
| Shallow 1 | Kapellkilen | 2017 | 59.666 N, 10.587 E | Eelgrass | 1.5 | 56 | 50 | 50 | 26 |
| Deep 1 | Kapellkilen | 2017 | 59.666 N, 10.587 E | Eelgrass | 2.5 | 56 | 100 | 70 | 35 |
| Deep 2 | Kapellkilen | 2017 | 59.666 N, 10.587 E | Eelgrass | 2.5 | 56 | 100 | 70 | 31 |
| Røvik 1 | Røvik | 2015 | 67.215 N, 15.008 E | Eelgrass | 1 | 50 | 50 | *unknown* | 25 |
| Røvik 2 | Røvik | 2015 | 67.215 N, 15.008 E | Eelgrass | 1 | 50 | 50 | *unknown* | 25 |
| Røvik 3 | Røvik | 2015 | 67.215 N, 15.008 E | Eelgrass | 1 | 50 | 50 | *unknown* | 25 |
| Porsanger 1 | Porsanger | 2022 | 70.112 N, 25.232 E | Eelgrass | 0.2 (intertidal) | 56 | 50 | 20 | 20 |
| Porsanger 2 | Porsanger | 2022 | 70.112 N, 25.232 E | Eelgrass | 0.2 (intertidal) | 56 | 50 | 10 | 10 |
| Porsanger 3 | Porsanger | 2022 | 70.112 N, 25.232 E | Eelgrass | 0.2 (intertidal) | 56 | 50 | 11 | 11 |

**Table S2. Environmental variables for each site. Note that for informative purposes mean dry bulk density (DBD) for each site is presented here, however the individual DBD of each core was used in the analysis. Temperature and salinity are surface values.**

| **Site name** | **Year** | **Habitat type** | **Sediment type** | **Wave exposure** | **Water depth (m)** | **Salinity** | **Temperature (°C): mean, minimum, maximum** | **Mean* dry bulk density (g cm^-3^)** | **Eelgrass aboveground biomass**  **(g dry weight m^-2^)** | **Eelgrass belowground biomass**  **(g dry weight m^-2^)** | **Eelgrass ag:bg ratio** |
| --- | --- | --- | --- | --- | --- | --- | --- | --- | --- | --- | --- |
| Sømskilen | 2017 | Eelgrass | Muddy sand | 6472 | 3 | 19.1 | 8.9  -1.0  21.3 | 0.25 | 215.2 | 66.9 | 3.22 |
| Sømskilen | 2017 | Unvegetated | Muddy sand | 6472 | 3 | 19.1 | 8.9  -1.0  21.3 | 0.31 | - | - | - |
| Ærøya | 2017 | Eelgrass | Coarse sand/gravel | 50606 | 3 | 25.3 | 9.1  0.01  21.2 | 1.55 | 44.3 | 93.2 | 0.48 |
| Ærøya | 2017 | Unvegetated | Coarse sand/gravel | 50606 | 3 | 25.3 | 9.1  0.01  21.2 | 1.60 | - | - | - |
| Merdø | 2017 | Eelgrass | Sand | 50958 | 3 | 26.8 | 8.9  -0.4  20.8 | 1.66 | 161.0 | 270.4 | 0.60 |
| Merdø | 2017 | Unvegetated | Sand | 50958 | 3 | 26.8 | 8.9  -0.4  20.8 | 1.66 | - | - | - |
| Langerompa | 2017 | Eelgrass | Mud | 12882 | 3 | 26.6 | 8.9  -0.4  20.9 | 0.39 | 350.9 | 97.4 | 3.60 |
| Langerompa | 2017 | Unvegetated | Coarse sand/gravel | 12882 | 3 | 26.6 | 8.9  -0.4  20.9 | 1.21 | - | - | - |
| Langerompa | 2017 | Eelgrass | Mud | 12882 | 7.5 | 26.6 | 8.9  -0.4  20.9 | 0.51 | 125.7 | 19.1 | 6.60 |
| Langerompa | 2017 | Unvegetated | Coarse sand/gravel | 12882 | 7.5 | 26.6 | 8.9  -0.4  20.9 | 1.62 | - | - | - |
| Hove | 2017 | Eelgrass | Fine mud | 1634 | 3 | 28.9 | 8.9  -1.7  21.5 | 0.13 | 483.2 | 166.2 | 2.91 |
| Hove | 2017 | Unvegetated | Mud | 1634 | 3 | 28.9 | 8.9  -1.7  21.5 | 0.11 | - | - | - |
| Sandspollen | 2018 | Unvegetated | Muddy sand | 1642 | 3.7 | 25.6 | 8.3  -1.5  22.3 | 0.51 | - | - | - |
| Sandspollen | 2018 | Unvegetated | Muddy sand | 1642 | 6 | 25.6 | 8.3  -1.5  22.3 | 0.84 | - | - | - |
| Kapellkilen | 2017 | Eelgrass | Muddy sand | 1481 | 1.5 | 25.6 | 8.3  -1.5  22.3 | 0.22 | 124.2 | 458.6 | 0.27 |
| Kapellkilen | 2017 | Eelgrass | Muddy sand | 1481 | 2.5 | 25.6 | 8.3  -1.5  22.3 | 0.16 | 298.7 | 312.1 | 0.96 |
| Kapellkilen | 2018 | Eelgrass | Muddy sand | 1481 | 3 | 25.6 | 8.3  -1.5  22.3 | 0.15 | not measured | not measured |  |
| Kapellkilen | 2018 | Unvegetated | Muddy sand | 1481 | 6 | 25.6 | 8.3  -1.5  22.3 | 1.43 | - | - | - |
| Røvik | 2015 | Eelgrass | Sand | 10012 | 1 | 28.5 | 6.4  -1.4  17.8 | 1.52 | 105.0 | 174.0 | 0.60 |
| Porsanger | 2022 | Eelgrass | Coarse sand/gravel | 22945 | 0.2  (inter-tidal) | 33.4 | 3.6  -1.9  16.5 | 1.82 | 18.1 | 19.0 | 0.14 |

**Figure S1. Correlation matrix of environmental variables in (a) eelgrass and (b) unvegetated sediments included in the PLS analysis**

1. **Eelgrass sediment**


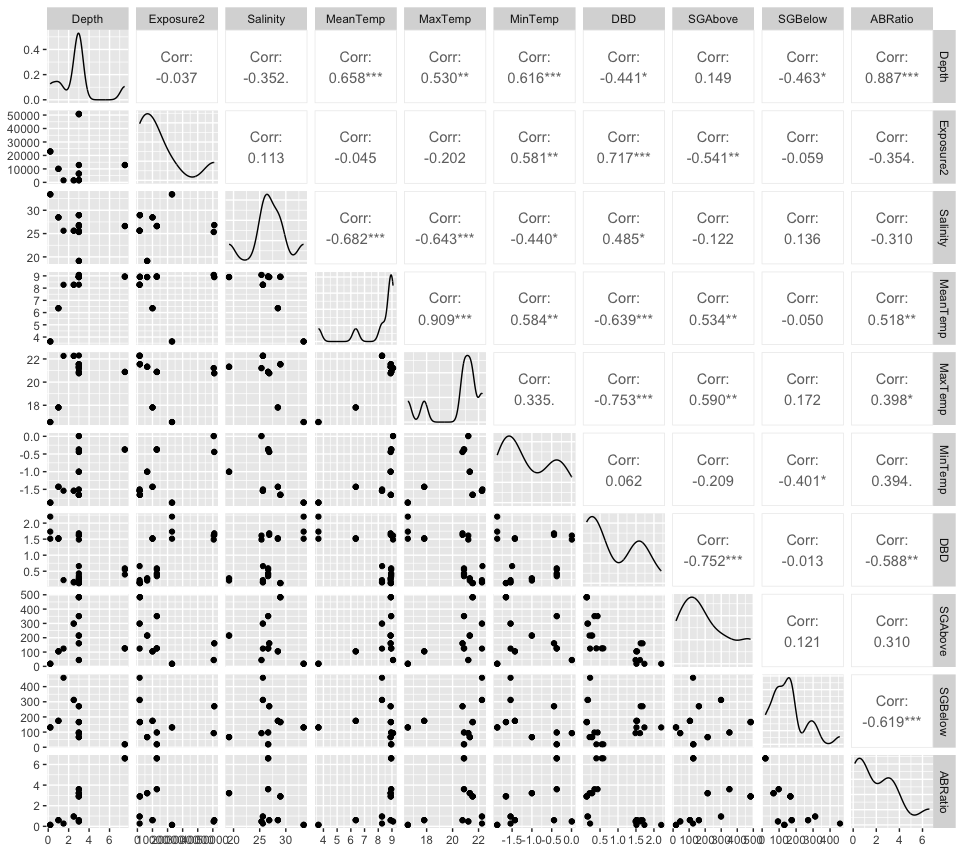


1. **Unvegetated sediment**

**
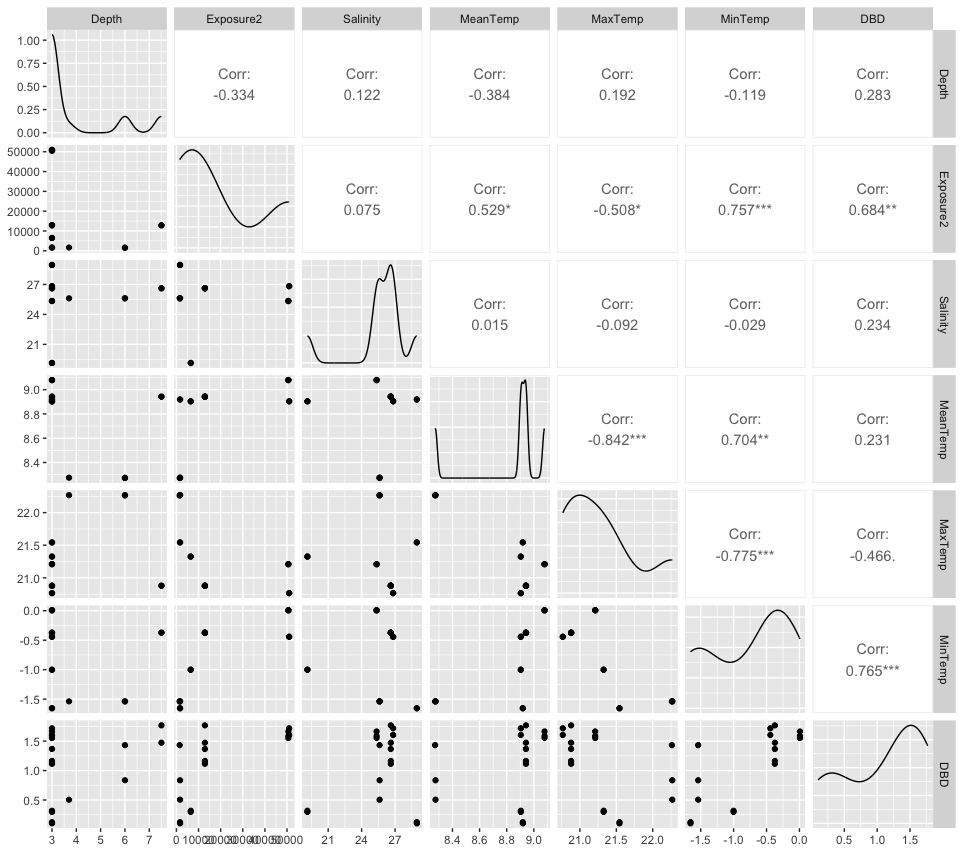
**

**Appendix 1. Methodology and reference list from literature review on eelgrass carbon stocks**

**Methods**

We did a comprehensive search of peer-reviewed publications published after 2010 in Web of Science and Google Scholar, using the search terms “(*seagrass* OR *eelgrass* OR *zostera*) AND *carbon*”. From this list, we selected all publications which contained data on organic carbon stocks or carbon densities in eelgrass (*Zostera* spp.) sediments. We included data on both natural and long-term (>4 years) restored meadows. In experimental or manipulative studies, we only used data from control treatments. We also excluded data from non-peer reviewed sources (e.g. theses, reports, etc.) though we note that there is likely a significant quantity of additional data on carbon stocks contained in “grey” literature that should be considered in a more comprehensive review.

We also checked the references of these publications for any publications we might have missed. We then extracted data, either from raw data in tables or supplementary material or from figures (using the program WebPlotDigitzer-4.5), at the site level (i.e. obtained a value for each site listed in the publication, which could be a mean or single value), and noted sediment depth and units. We included data given as carbon stock per area (e.g. g C m^-2^) or as carbon density per volume (e.g. g C cm^-3^) if the sediment volume was provided and could thus be converted to carbon per area. All data were converted to a standard unit of carbon stock (Mg C ha^-1^) for the top 1 m of sediment, as described in the methods section of the main text.

Some data points were represented in multiple publications, and we filtered these out to avoid duplicates. In total, we obtained 385 data points from 54 publications, which included 315 data points on *Zostera marina*, 23 on *Z. noltii,* 16 on mixed *Z. marina/Z. noltii*, 9 on *Z. japonica,* 9 on *Z. muelleri*, 6 on mixed *Z. marina/Z. japonica*, 3 on *Z. capensis*, 1 each on *Z. caulescens, Z. chilensis, Z. nigricaulis*, mixed *Z. muelleri/Z.nigricaulis,* and mixed *Z. tasmanica/Z. muelleri/Z. mucronata*.

**Reference list for literature review**

1. Advani, S. & Satterfield, T. Attributions of cause of oyster mortality on the British Columbia coast: Oyster growers’ and scientists’ perspectives. Ocean & Coastal Management 251, 107066 (2024).

2. Aoki, L. R. et al. Seagrass recovery following marine heat wave influences sediment carbon stocks. Front. Mar. Sci. 7, 576784 (2021).

3. Barañano, C., Fernández, E. & Méndez, G. Clam harvesting decreases the sedimentary carbon stock of a Zostera marina meadow. Aquat. Bot. 146, 48–57 (2018).

4. Billman, M., Santos, I. R. & Jahnke, M. Small carbon stocks in sediments of Baltic Sea eelgrass meadows. Front. Mar. Sci. 10, 1219708 (2023).

5. Bulmer, R. H. et al. Blue carbon stocks and cross-habitat subsidies. Front. Mar. Sci. 7, 380 (2020).

6. Cuellar-Martinez, T., Ruiz-Fernández, A. C., Sanchez-Cabeza, J.-A., Pérez-Bernal, L.-H. & Sandoval-Gil, J. Relevance of carbon burial and storage in two contrasting blue carbon ecosystems of a north-east Pacific coastal lagoon. Sci. Total Environ. 675, 581–593 (2019).

7. Dahl, M. et al. High seasonal variability in sediment carbon stocks of cold‐temperate seagrass meadows. J. Geophys. Res. Biogeosci. 125, e2019JG005430 (2020).

8. Dahl, M. et al. Sediment properties as important predictors of carbon storage in *Zostera marina* meadows: a comparison of four European areas. PLoS ONE 11, e0167493 (2016).

9. Dahl, M. et al. Increased current flow enhances the risk of organic carbon loss from *Zostera marina* sediments: insights from a flume experiment. Limnol. Oceanogr. 63, 2793–2805 (2018).

10. de la Cerda-Marín, M. del C. et al. Blue carbon stock in *Zostera marina* meadows in the Ría de Ferrol (NW Iberian Peninsula). Span. J. Soil Sci. 13, 11352 (2023).

11. do Amaral Camara Lima, M., Ward, R. D., Joyce, C. B., Kauer, K. & Sepp, K. Carbon stocks in southern England’s intertidal seagrass meadows. Estuar. Coast. Shelf Sci. 275, 107947 (2022).

12. Douglas, T. J., Schuerholz, G. & Juniper, S. K. Blue carbon storage in a northern temperate estuary subject to habitat loss and chronic habitat disturbance: Cowichan Estuary, British Columbia, Canada. Front. Mar. Sci. 9, 857586 (2022).

13. Ewers Lewis, C. J., Carnell, P. E., Sanderman, J., Baldock, J. A. & Macreadie, P. I. Variability and vulnerability of coastal ‘blue carbon’ stocks: a case study from southeast Australia. Ecosystems 21, 263–279 (2018).

14. Ewers Lewis, C. J. et al. Drivers and modelling of blue carbon stock variability in sediments of southeastern Australia. Biogeosciences 17, 2041–2059 (2020).

15. Fourqurean, J. W. et al. Seagrass ecosystems as a globally significant carbon stock. Nat. Geosci. 5, 505–509 (2012).

16. Fu, C. et al. Stocks and losses of soil organic carbon from Chinese vegetated coastal habitats. Glob. Change Biol. 27, 202–214 (2021).

17. Green, A., Chadwick, M. A. & Jones, P. J. S. Variability of UK seagrass sediment carbon: implications for blue carbon estimates and marine conservation management. PLoS ONE 13, e0204431 (2018).

18. Greiner, J. T., McGlathery, K. J., Gunnell, J. & McKee, B. A. Seagrass restoration enhances “blue carbon” sequestration in coastal waters. PLoS ONE 8, e72469 (2013).

19. Holmer, M., Carta, C. & Andersen, F. Ø. Biogeochemical implications for phosphorus cycling in sandy and muddy rhizosphere sediments of Zostera marina meadows (Denmark). Mar. Ecol. Prog. Ser. 320, 141–151 (2006).

20. Jankowska, E., Michel, L. N., Zaborska, A. & Włodarska-Kowalczuk, M. Sediment carbon sink in low-density temperate eelgrass meadows (Baltic Sea). J. Geophys. Res. Biogeosci. 121, 2918–2934 (2016).

21. Kauffman, J. B. et al. Total ecosystem carbon stocks at the marine‐terrestrial interface: blue carbon of the Pacific Northwest Coast, United States. Glob. Change Biol. 26, 5679–5692 (2020).

22. Kennedy, H. et al. Species traits and geomorphic setting as drivers of global soil carbon stocks in seagrass meadows. Glob. Biogeochem. Cy. 36, e2022GB007481 (2022).

23. Kim, S. H. et al. Variability in blue carbon storage related to biogeochemical factors in seagrass meadows off the coast of the Korean peninsula. Sci. Total Environ. 813, 152680 (2022).

24. Kindeberg, T., Ørberg, S. B., Röhr, M. E., Holmer, M. & Krause-Jensen, D. Sediment stocks of carbon, nitrogen, and phosphorus in Danish eelgrass meadows. Front. Mar. Sci. 5, 474 (2018).

25. Krause-Jensen, D. et al. Nordic blue carbon ecosystems: status and outlook. Front. Mar. Sci. 9, 847544 (2022).

26. Lange, T. et al. Large-scale eelgrass transplantation: a measure for carbon and nutrient sequestration in estuaries. Mar. Ecol. Prog. Ser. 685, 97–109 (2022).

27. Lavery, P. S., Mateo, M.-Á., Serrano, O. & Rozaimi, M. Variability in the carbon storage of seagrass habitats and its implications for global estimates of blue carbon ecosystem service. PLoS ONE 8, e73748 (2013).

28. Lei, J. et al. Spatial heterogeneity in sediment and carbon accretion rates within a seagrass meadow correlated with the hydrodynamic intensity. Sci. Total Environ. 854, 158685 (2023).

29. Leiva‐Dueñas, C. et al. Capturing of organic carbon and nitrogen in eelgrass sediments of southern Scandinavia. Limnol. Oceanogr. 68, 631–648 (2023).

30. Marbà, N., Krause-Jensen, D., Masqué, P. & Duarte, C. M. Expanding Greenland seagrass meadows contribute new sediment carbon sinks. Sci. Rep. 8, 14024 (2018).

31. Mazarrasa, I. et al. Drivers of variability in blue carbon stocks and burial rates across European estuarine habitats. Sci. Total Environ. 886, 163957 (2023).

32. McGlathery, K. J. et al. Recovery trajectories during state change from bare sediment to eelgrass dominance. Mar. Ecol. Prog. Ser. 448, 209–221 (2012).

33. Miyajima, T. et al. Geographic variability in organic carbon stock and accumulation rate in sediments of East and Southeast Asian seagrass meadows. Glob. Biogeochem. Cycles 29, 397–415 (2015).

34. Moksnes, P. et al. Major impacts and societal costs of seagrass loss on sediment carbon and nitrogen stocks. Ecosphere 12, e03658 (2021).

35. Novak, A. B. et al. Factors influencing carbon stocks and accumulation rates in eelgrass meadows across New England, USA. Estuar. Coast. 43, 2076–2091 (2020).

36. Oreska, M. P. J., McGlathery, K. J. & Porter, J. H. Seagrass blue carbon spatial patterns at the meadow-scale. PLoS ONE 12, e0176630 (2017).

37. Oreska, M. P. J., Wilkinson, G. M., McGlathery, K. J., Bost, M. & McKee, B. A. Non-seagrass carbon contributions to seagrass sediment blue carbon. Limnol. Oceanogr. 63, 33-S18 (2018).

38. Poppe, K. L. & Rybczyk, J. M. Carbon sequestration in a Pacific Northwest eelgrass (*Zostera marina*) meadow. Northwest Sci. 92, 80–91 (2018).

39. Postlethwaite, V. R., McGowan, A. E., Kohfeld, K. E., Robinson, C. L. K. & Pellatt, M. G. Low blue carbon storage in eelgrass (*Zostera marina*) meadows on the Pacific coast of Canada. PLoS ONE 13, e0198348 (2018).

40. Potouroglou, M. et al. The sediment carbon stocks of intertidal seagrass meadows in Scotland. Estuar. Coast. Shelf Sci. 258, 107442 (2021).

41. Prentice, C. et al. A synthesis of blue carbon stocks, sources, and accumulation rates in eelgrass (*Zostera marina*) meadows in the northeast Pacific. Glob. Biogeochem. Cy. 34, (2020).

42. Prentice, C., Hessing‐Lewis, M., Sanders‐Smith, R. & Salomon, A. K. Reduced water motion enhances organic carbon stocks in temperate eelgrass meadows. Limnol. Oceanogr. 64, 2389–2404 (2019).

43. Qin, L.-Z., Suonan, Z., Kim, S. H. & Lee, K.-S. Coastal sediment nutrient enrichment alters seagrass blue carbon sink capacity. Environ. Sci. Technol. 55, 15466–15475 (2021).

44. Röhr, M. E., Boström, C., Canal-Vergés, P. & Holmer, M. Blue carbon stocks in Baltic Sea eelgrass (*Zostera marina*) meadows. Biogeosciences 13, 6139–6153 (2016).

45. Röhr, M. E. et al. Blue carbon storage capacity of temperate eelgrass (Zostera marina) meadows. Glob. Biogeochem. Cycles 32, 1457–1475 (2018).

46. Román, M. et al. Loss of surficial sedimentary carbon stocks in seagrass meadows subjected to intensive clam harvesting. Mar. Environ. Res. 175, 105570 (2022).

47. Saavedra-Hortua, D., Nagelkerken, I., Estupinan-Suarez, L. M. & Gillis, L. G. Effects of connectivity on carbon and nitrogen stocks in mangrove and seagrass ecosystems. Sci. Total Environ. 896, 164829 (2023).

48. Sousa, A. I., da Silva, J. F., Azevedo, A. & Lillebø, A. I. Blue carbon stock in *Zostera noltei* meadows at Ria de Aveiro coastal lagoon (Portugal) over a decade. Sci. Rep. 9, 14387 (2019).

49. Stevenson, A., Ó Corcora, T. C., Hukriede, W., Schubert, P. R. & Reusch, T. B. H. Substantial seagrass blue carbon pools in the southwestern Baltic Sea include relics of terrestrial peatlands. Front. Mar. Sci. 9, 949101 (2022).

50. Ward, M. A. et al. Blue carbon stocks and exchanges along the California coast. Biogeosciences 18, 4717–4732 (2021).

51. Wasserman, J., Human, L. R. D. & Adams, J. B. Blue carbon stocks in southern Africa’s endangered seagrass Zostera capensis. Estuar. Coast. Shelf Sci. 284, 108296 (2023).

52. Watanabe, K. & Kuwae, T. An unintended ecological benefit from human intervention: the enhancement of carbon storage in seagrass meadows. J. Appl. Ecol. 58, 2441–2452 (2021).

53. Watanabe, K., Seike, K., Kajihara, R., Montani, S. & Kuwae, T. Relative sea‐level change regulates organic carbon accumulation in coastal habitats. Glob. Change Biol. 25, 1063–1077 (2019).

54. Yue, S. et al. The super typhoon Lekima (2019) resulted in massive losses in large seagrass (*Zostera japonica*) meadows, soil organic carbon and nitrogen pools in the intertidal Yellow River Delta, China. Sci. Total Environ. 793, 148398 (2021).
